# Identifying genomic surveillance gaps in Africa for the global public health response to West Nile virus: a systematic review

**DOI:** 10.1016/j.lanmic.2025.101176

**Published:** 2025-07-24

**Authors:** Monika Moir, Nikita Sitharam, Laura Marije Hofstra, Graeme Dor, Gaspary Mwanyika, Yajna Ramphal, Martina L Reichmuth, James Emmanuel San, Robert Gifford, Eduan Wilkinson, Derek Tshiabuila, Wolfgang Preiser, Abla Ahouefa Konou, Molalegne Bitew, Anyebe Bernard Onoja, Giacomo Maria Paganotti, Adugna Abera, James Ayei Maror, John Kayiwa, Sara Abuelmaali, Eddy Kinganda Lusamaki, CLIMADE Consortium, Marietjie Venter, Felicity Burt, Cheryl Baxter, Richard Lessells, Tulio de Oliveira, Houriiyah Tegall

**Affiliations:** Centre for Epidemic Response and Innovation, School for Data Science and Computational Thinking, Stellenbosch University, Matieland, South Africa; Centre for Epidemic Response and Innovation, School for Data Science and Computational Thinking, Stellenbosch University, Matieland, South Africa; Centre for Epidemic Response and Innovation, School for Data Science and Computational Thinking, Stellenbosch University, Matieland, South Africa; Centre for Epidemic Response and Innovation, School for Data Science and Computational Thinking, Stellenbosch University, Matieland, South Africa; Centre for Epidemic Response and Innovation, School for Data Science and Computational Thinking, Stellenbosch University, Matieland, South Africa; Department of Applied Sciences, Mbeya University of Science and Technology, Mbeya, Tanzania; Centre for Epidemic Response and Innovation, School for Data Science and Computational Thinking, Stellenbosch University, Matieland, South Africa; Institute of Social and Preventive Medicine, University of Bern, Bern, Switzerland; Duke Human Vaccine Institute, Duke University, Durham, NC, USA; School of Laboratory Medicine and Medical Sciences, University of KwaZulu-Natal, Durban, South Africa; Centre for Epidemic Response and Innovation, School for Data Science and Computational Thinking, Stellenbosch University, Matieland, South Africa; Centre for Epidemic Response and Innovation, School for Data Science and Computational Thinking, Stellenbosch University, Matieland, South Africa; Centre for Epidemic Response and Innovation, School for Data Science and Computational Thinking, Stellenbosch University, Matieland, South Africa; Division of Medical Virology, Faculty of Medicine and Health Sciences, Stellenbosch University, Matieland, South Africa; National Health Laboratory Service, Tygerberg Campus, Cape Town, South Africa; Laboratoire de Biologie moléculaire et d’Immunologie (BIOLIM/FSS-UL), Université de Lomé, Lomé, Togo; Bio and Emerging Technology Institute, Addis Ababa, Ethiopia; Department of Virology, University of Ibadan, Ibadan, Nigeria; Botswana-University of Pennsylvania Partnership, Gaborone, Botswana; Division of Infectious Diseases, Perelman School of Medicine, University of Pennsylvania, Philadelphia, PA, USA; Ethiopian Public Health Institute, Addis Ababa, Ethiopia; National Public Health Laboratory, Ministry of Health, Juba, South Sudan; Uganda Virus Research Institute, Entebbe, Uganda; National Public Health Laboratory, Khartoum, Sudan; Institut National de Recherche Biomédicale, Kinshasa, Democratic Republic of the Congo; Service de Microbiologie, Département de Biologie Médicale, Université de Kinshasa, Kinshasa, Democratic Republic of the Congo; TransVIHMI, Université de Montpellier, INSERM, IRD, Montpellier, France; Emerging Viral Threat, One Health Surveillance and Vaccines Division, Infectious Diseases and Oncology Research Institute, University of the Witwatersrand, Johannesburg, South Africa; Centre for Emerging Arbo and Respiratory Virus Research, Department Medical Virology, Faculty of Health, University of Pretoria, Pretoria, South Africa; Division of Virology, National Health Laboratory Services, Universitas Hospital, Bloemfontein, South Africa; Pathogen Research Laboratory, Division of Virology, University of the Free State, Bloemfontein, South Africa; Centre for Epidemic Response and Innovation, School for Data Science and Computational Thinking, Stellenbosch University, Matieland, South Africa; KwaZulu-Natal Research Innovation and Sequencing Platform, University of KwaZulu-Natal, Durban, South Africa; Centre for Epidemic Response and Innovation, School for Data Science and Computational Thinking, Stellenbosch University, Matieland, South Africa; KwaZulu-Natal Research Innovation and Sequencing Platform, University of KwaZulu-Natal, Durban, South Africa; Centre for Epidemic Response and Innovation, School for Data Science and Computational Thinking, Stellenbosch University, Matieland, South Africa

## Abstract

West Nile virus (WNV) is a priority pathogen that poses a high risk for public health emergencies of global concern. Although WNV is endemic to Africa, only few (n=63) whole genomic sequences are available from the continent. In this Review, we examined the status of the molecular testing and genomic sequencing of WNV across Africa and mapped its global spatiotemporal spread. WNV has been detected in 39 African countries, the Canary Islands, and Réunion Island. Although publications, including those with molecular data, originated from 24 of these countries, genomic sequences were available from only 16 countries. Our analysis identified regions with detected viral circulation but without molecular surveillance. The current literature has substantial knowledge gaps in terms of the disease burden, molecular epidemiology, and distribution of WNV in Africa. Addressing these gaps requires an integrated One Health surveillance approach, which is challenging to establish. We propose three key surveillance needs that could improve the current understanding of the WNV disease burden in Africa, to strengthen the global public health response to this vector-borne disease.

## Introduction

West Nile virus (WNV; *Orthoflavivirus nilense*), a virus of One Health importance, is present in a transmission cycle between birds and vector-competent mosquitoes, primarily of the *Culex* genus. WNV can also be transmitted incidentally to dead-end hosts such as humans, horses, and other animal species.^1,2^ WNV was first isolated in 1937 from a patient with fever in Uganda.^3^ In humans, 80% of WNV infections are asymptomatic; symptomatic infections typically present as a mild febrile illness with headaches, myalgia, arthralgia, and rash. Neuroinvasive diseases occur in less than 1% of cases, with a fatality rate of 10–30%.^4^

WNV is a WHO priority pathogen that poses a high risk for public health emergencies of global concern.^5^ Over the past two decades, WNV has emerged as a public health concern in the Americas and Europe, causing neurological disease^6^ and expanding its geographical range.^7^ Considering the widespread distribution of WNV and the severity of its outbreaks in immunologically naive populations, understanding the global historical dispersal of the virus from its inferred African origin is crucial.^8,9^ However, phylogeographic reconstructions conducted are restricted to only some regions of the world.^10–12^

Although WNV was first detected in Africa and is endemic to the continent,^13^ and numerous African countries experience a long-term disease burden due to the virus, the true transmission risk in the continent is often unclear, with insufficient epidemiological data for 19 countries.^8^ Genomic data are crucial for investigating transmission dynamics and understanding disease outbreaks.^9,14,15^ Considering the power of molecular epidemiology,^16,17^ a full assessment of the landscape of molecular surveillance for WNV is essential. Therefore, in this Review, we assessed the extent of PCR testing and genomic sequencing of WNV across Africa. We identified areas where the circulation of WNV is confirmed but molecular data are insufficient and increased molecular surveillance could be beneficial. In addition, we reconstructed the spatiotemporal spread of the virus within Africa and globally.

## Methods

### Review of genomic sequencing and PCR testing in Africa

This systematic review assessed the landscape of molecular surveillance of WNV in Africa. The study was carried out according to PRISMA criteria^18^ and registered with PROS-PERO (CRD42024614647). The publications were obtained on April 29, 2024, using a search strategy described in the appendix (p 1).

Following the removal of duplicates, two independent reviewers (MM, NS) screened the publications (n=334). The criteria used to select publications for full-text screening included: the WNV infections reported were natural (excluded experimental infections); the nucleic acid data were produced or analysed via PCR or genomic sequencing (publications that used a combination of molecular diagnostic techniques were also included); and the samples were collected in Africa. Publications with data from islands (Canary Islands, Réunion Island, Mayotte, and Tromelin Island) geographically close to the continent but not politically classified as African nations were also included in the study, as birds move between these regions,^19,20^ with potential for viral transmission between them.

Two independent reviewers (MM, NS) extracted the relevant data (and checked the accuracy of the extractions) from the included publications (n=79; appendix p 1). The extracted data are available from the linked GitHub repository. Studies were classified as: focused on an African country; focused elsewhere but analysed sequences from Africa; or focused elsewhere but produced or analysed sequences, or both, from Africa.

The publications were categorised into a study type on the basis of the following definitions. Indicator-based surveillance (from the WHO African region’s technical guidelines for integrated disease surveillance and response)^21^ involved regular identification, collection, and monitoring of data from formal sources, such as facility-based, sentinel, and syndromic surveillance. Event-based surveillance involved events of potential risk to public health, such as unusual disease, and events in the community or environment causing potential exposure to disease.^21^ Case studies involved clinical case reports of patients with classic or unusual disease presentations.^22^ Travel-related surveillance involved a case of a traveller entering or departing from an African country. Research involved studies on transmission, characterisation, diversity, etc, which have not been conducted as part of the surveillance strategies mentioned earlier in this paragraph.

The sequence information extracted from the publications (n=257 sequences) was cross-referenced with sequences publicly available in the National Center for Biotechnology Information database (n=289; appendix pp 2–3).

### Detection of WNV from cases, deaths, seroprevalence surveys, and other research studies

To compare the locations in which molecular studies have been conducted and those of viral records (from reported cases, seroprevalence surveys, and other research studies), we used the *West Nile fever: global status* 2023 report by the Global Infectious Diseases and Epidemiology Network^23^ as a guide to find available literature. The primary sources referenced in this report were reviewed to identify viral records with fine-scale location data.

The data extracted from the publications included the year of study, host species, number of reported cases or deaths, seroprevalence results, and tests performed to ascertain WNV positivity. Non-systematic searches were also performed for additional reports not listed in the Global Infectious Diseases and Epidemiology Network report. When mapping the total number of viral occurrences relative to molecular sampling locations, all records were filtered to retain only one occurrence per location point. The same approach was followed for the molecular studies. Data visualisations were produced with RStudio 2024.04.2+764 and QGIS 3⋅26⋅3. If detailed location data were not available for plotting, the centroid position of the country was used.

### Phylogenetic analyses

Whole genomes (>10 kilobase length) were retrieved from NCBI Virus on Aug 13, 2024. Sequences were analysed using the West Nile Virus Typing Tool by Genome Detective, and lineage 1A (L1A) and lineage 2 (L2) sequences were selected for further analysis. The final nucleotide alignments contained 258 sequences for L1A and 541 sequences for L2. Maximum likelihood phylogenies were constructed using IQTree^24^ version 2.3.6 and 1000 bootstrap approximations. Trees were inspected for temporal molecular clock signals using the clock functionality of TreeTime^25^ and outlier sequences were removed. Time-scaled phylogenies were created with tip-dating calibration and adjusted mutation rates for each lineage (β=4⋅35×10^−4^ for L1A and β=2⋅62×10^−4^ for L2; appendix p 3). The mugration model in TreeTime was run, and a custom Python script was used for the spatiotemporal dispersal analysis. Detailed methodology for phylogenetic analyses is discussed in the appendix (p 4).

## Results

### Molecular surveillance for WNV in Africa is mostly driven by research

**Figure 1:**
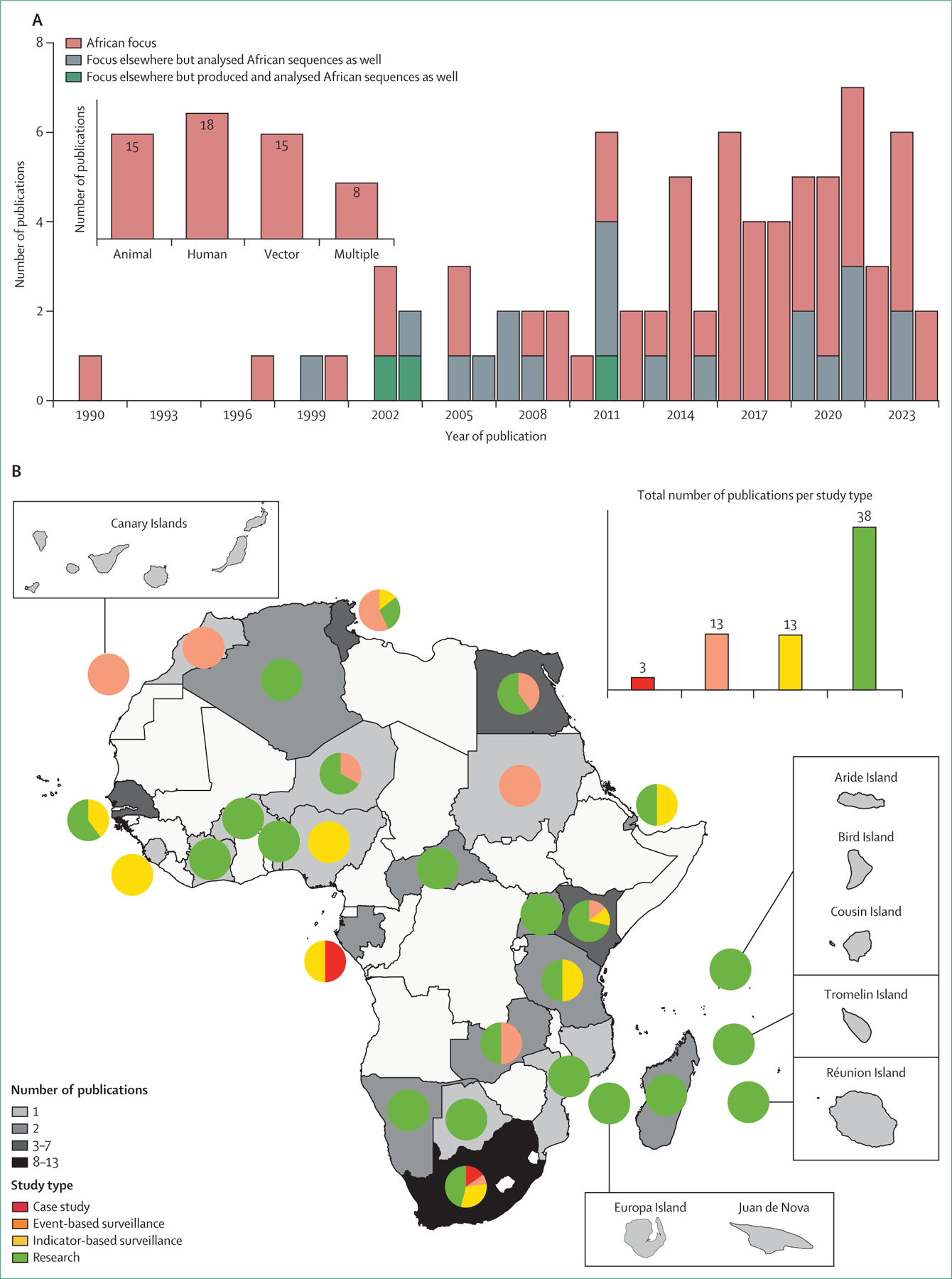
Publications of West Nile virus genomics in Africa and distribution of study types (A) Number of publications that used molecular methods. Studies are categorised on the basis of whether the geographical focus is Africa or elsewhere and whether the molecular data are produced or analysed, or both, from African samples. The inset graph depicts, for studies with an African focus, the total number of studies per host or vector group. Multiple indicates two or more host or vector groups. (B) Publications with an African focus have been mapped with pie charts to show proportions of study types per country. The shading gradient reflects the total number of studies. The inset graph depicts publications per study type. Islands are zoomed in peripheral boxes.

Publications were retrieved from PubMed (n=248) or Scopus (n=263); additional publications (n=3) were identified by going through the references of articles that were included in the review after the initial search. 79 of these publications were found to have used molecular methods to study WNV in Africa since 1990, with a peak in the publication rate observed in 2021 (figure 1). The earliest publication reported the isolation of WNV from patients with hepatitis in Bangui, Central African Republic.^26^ Of the 79 publications, 56 focused on Africa, whereas 23 focused outside Africa. Of those 23, three generated African genomic data and 20 used African sequences in their analyses (figure 1). Of the 56 studies focused on Africa, 18 used human samples, 15 used animal host samples (birds, horses, livestock, and wildlife), 15 used vector samples (mosquitoes and ticks), and eight screened multiple host or vector groups.

The studies focused on Africa consisted of publications from 24 countries in continental Africa, with most (n=23) originating from South Africa, Senegal, and Tunisia (figure 1). The majority were research studies (n=38) conducted across most of the continent. Case studies (n=3) were published from Gabon and South Africa; event-based surveillance studies (n=13) from eight countries and the Canary Islands; and indicator-based surveillance studies (n=13) from nine countries. No travel-related studies were identified. 31 of the 55 African countries did not publish any molecular studies.

### Molecular testing for WNV remains low in humans

This study collated WNV infections confirmed by means of PCR or sequencing, which are indicative of active infections, for human cases (table 1), animal hosts (table 2), and vectors (appendix pp 4–7).

Molecular screening studies for WNV in humans were available for 12 countries, of which eight reported positive detections (table 1). Studies from Tunisia showed the highest percentage positivity, as these studies included individuals with suspected infection or confirmed serology. Tunisia had repeated outbreaks in 1997,^57^ 2003, 2012, and 2018.^58^ Using molecular testing and IgM serology, two cohort studies diagnosed 3⋅65% of individuals with WNV infection in Gauteng and 9⋅07% in Gauteng and Mpumalanga, South Africa.^40^ Another study from South Africa detected WNV via PCR and sequencing in one cerebrospinal fluid sample from individuals with suspected WNV neurological infection but revealed positive serology in 19⋅4% of the cohort.^31^ Since these studies were conducted outside of recognised outbreak periods, the findings highlight that the WNV disease burden in South Africa is still underestimated.^31,40^ Upon testing patients with fever, PCR-positive infections were also found in Tanzania,^37,38^ Nigeria,^41^ Senegal,^35,45^ and Sierra Leone,^33^ in addition to one case study in Gabon.^46^ WNV was not detected in human studies in Burkina Faso,^44^ Egypt,^36^ and Kenya.^32^

### WNV has a broad animal host range with low detection rates

WNV nucleic acids were detected from 35 avian species in Africa (table 2). The majority of these species were sampled from the Tana River and Garissa in Kenya, a known stopover for birds migrating from northern Europe to southern Africa.^47^ WNV was isolated from a long-billed crombec, a sentinel pigeon, and an ostrich in South Africa.^27^ WNV was also detected in a marsh tchagra from Central African Republic,^48^ in addition to a chicken^50^ and a greater vasa parrot from Madagascar.^48^ All positive detections were outside of known outbreak periods, except for one mallard duck sampled in Tunisia around the time of the 2012 outbreak in humans.

Horses are sentinel animals useful for estimating the risk of human infections.^51^ However, molecular studies in horses are available for only two countries (table 2): South Africa^51^ (the only country with evidence of active surveillance for infectious pathogens in horses) and Morocco (an outbreak of L1 infections in 2003).

All WNV detections in livestock and wildlife were from southern Africa, except for L1 infection in a bushbaby from Senegal (table 2). WNV shows broad host tropism, with detection in several species of livestock, domestic animals, and wildlife.

### WNV nucleic acids were detected in 27 vector species

In the appendix (pp 4–7), we present a synthesis of WNV detections from 13 countries in six mosquito genera (*Aedeomyia*, *Aedes*, *Anopheles*, *Culex*, *Mansonia*, and *Mimomyia*) and two tick vectors (*Amblyomma gemma* and *Rhipicephalus pulchellus*). The lowest minimum infection rate (an estimate of the proportion of infected mosquitoes in a population) of 0⋅01 was from sandfly vectors in Tasnala, Niger, whereas the greatest minimum infection rate of 23⋅00 was from *Culex perexiguus* in Egypt’s Nile Valley. WNV was detected in its primary vectors: *Culex pipiens* sensu stricto, *Culex quinquefasciatus*, and *Culex univittatus*, in Djibouti, Egypt, Madagascar, Namibia, Senegal, South Africa, Tunisia, and Zambia.

### 127 subnational administrative locations showed WNV circulation but an absence of molecular surveillance

**Figure 2:**
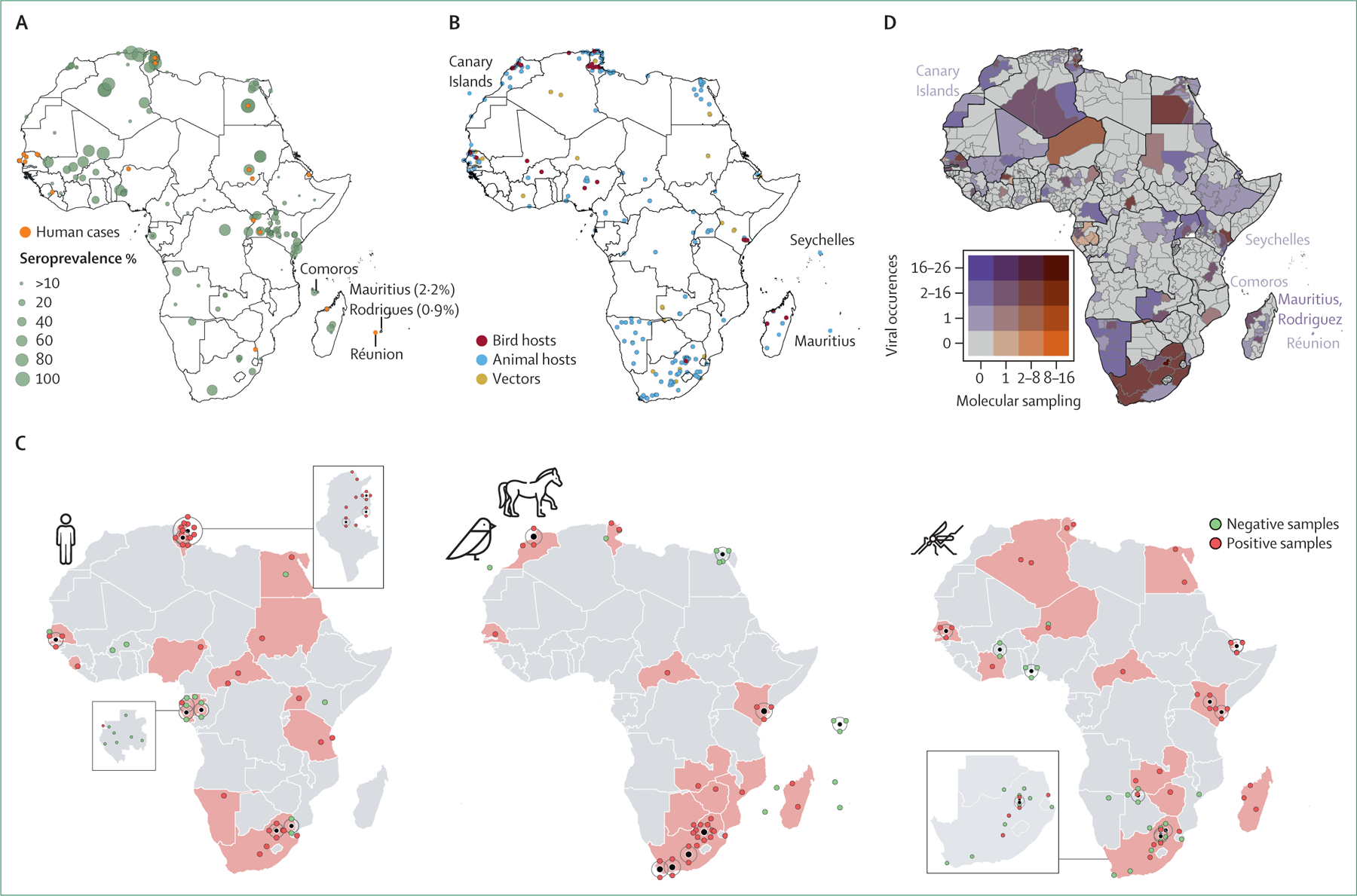
Circulation and molecular surveillance of WNV in Africa (A) Geolocations of WNV cases in humans (orange) and prevalence from serological surveys (green circles, percentage represented by circle size). (B) Georeferenced locations of viral detections (cases, deaths, seroprevalence) in birds (red), dead-end animal hosts (blue), and mosquito and tick vectors (gold). (C) Geographical distribution of molecular detections of WNV (PCR and sequencing) from humans, animals, and vectors. Red indicates countries from which positive samples were collected. Point locations are shown with a point displacement method, in which the locality of overlapping points is represented with a black marker and the number of points at this location is represented with coloured dots on a ring around the central marker. If details about the locations were not available, then points were plotted in the centre of the country. (D) Bivariate map depicting WNV circulation (total number of viral occurrences) and the total number of molecular sampling locations on a subnational administration level. Locations were not mapped wherever they did not have detailed spatial information. The colour used for the island names matches their designation on the bivariate colour scale. WNV=West Nile virus.

WNV occurrences were collated from reported cases, seroprevalence surveys, and other research studies. We used 72 literature sources to geolocate WNV detections in humans from 29 continental African countries, Comoros, Mauritius, Rodrigues, and Réunion Island. WNV was detected in humans in all African regions, with high seroprevalence (>60%) in Algeria, Tunisia, Mali, Nigeria, Egypt, Sudan, the Democratic Republic of the Congo, Uganda, and Kenya (figure 2). Since 1937, 301 human cases have been reported from ten African countries and Réunion Island. WNV was detected in birds in ten African countries (figure 2). For other animal species, evidence of WNV was found in 22 African countries, from 62 literature sources (figure 2). 25 literature sources were used to gather information on detections from vectors for 13 African countries (figure 2). Considering all host and vector species, WNV has been detected in 39 African countries (including Comoros, Seychelles, and Mauritius), the Canary Islands, and Réunion Island.

Our review of molecular studies shows PCR detection of WNV from 13 African countries and seven western Indian Ocean islands (appendix p 7). Studies from 20 African countries and the Canary Islands involved sample collection for genomic sequencing (appendix p 7). Tunisia (n=8) and South Africa (n=13) showed the greatest extent of study locations. Many countries had sparse sampling locations. Of note, a 225-nucleotide sequence (OL790153) from Zimbabwe did not have location information, and a preprint reported detections from crocodiles and mosquitoes.^59^

Active WNV infections detected via PCR testing or genomic sequencing, or both, in 21 African countries, across all regions, for humans, animals, and vectors have been represented in figure 2. Most studies from Tunisia involved testing of human samples with positive detections in the northwestern regions,^28,39,42,43^ and little testing has been reported in the south. Human cases (figure 2) without molecular data were reported from Djibouti^60^ and Madagascar.^61^ A representation of the temporal range of publications and the number of publicly available sequences per country is given in the appendix (p 8). Senegal, South Africa, and Tunisia show comparatively high study and sequencing efforts, whereas Central African Republic, the Democratic Republic of the Congo, Egypt, Ethiopia, and Uganda have few published sequences.

Viral circulation (total number of occurrences) was also linked with the total number of molecular sampling locations on a subnational administrative level in this study (figure 2). Such an analysis identified 127 administrative-level locations and six islands and island archipelagos with viral circulation but without sufficient molecular surveillance, particularly many regions of Namibia, Ethiopia, Morocco, Western Sahara, and Mali. Regions with high WNV circulation and a higher number of molecular sampling locations included seven provinces of South Africa, Garissa County of Kenya, and Monastir in Tunisia.

Birds play a crucial role in the transmission of WNV; thus, humans in areas where birds spend an extended period face a risk of spillover.^62^ Key biodiversity areas (KBAs) harbour substantial regional biodiversity^63^ and consist of important bird and biodiversity areas identified by BirdLife International.^63^ We identified regions with confirmed WNV detections that overlap with KBAs^63^ and areas of high human population density^64^ that could be susceptible to viral spillovers. These areas included the Eastern Cape of South Africa, western and northwestern provinces of Zambia, Afar and Oromia states of Ethiopia, four governates of Egypt (Qena, Monufia, Kafr el-Sheikh, and Al Sharqi), central and eastern regions of Cameroon, and five regions of Morocco (Souss-Massa, Casablanca-Settat, Marrakech-Safi, Fez-Meknes, and Béni Mellal-Khénifra). Given the genetic diversity of WNV and the high human density and numerous KBAs in west Africa, surveillance across the region would most likely yield valuable insights. Similarly, nationwide surveillance is important in Uganda; although WNV was first isolated in Uganda, the country has published only three genomes since. Research in Ethiopia and Kenya’s Great Rift Valley could also be valuable owing to the diverse bird species found in these regions and potential for human–animal contact (figure 2).

### Africa has produced the third-highest number of whole genomes of WNV

Publicly available genomes were used to assess the temporal and spatial distributions of WNV lineages in this study. Globally, Africa has the third-highest number of whole genomes (n=63) of WNV (appendix p 9). Considering this finding, we assessed the laboratory infrastructure used in generating these data. Wet laboratory procedures were performed within the respective African countries for most studies 40 (67⋅8%) of 59, with 17 (28⋅8%) of 59 using an external laboratory (appendix p 9). 18 (30⋅5%) of 59 studies used solely RT-PCR, whereas 41 (69⋅5%) of 59 used sequencing, mainly of the non-structural protein 5 and envelope genes (appendix p 10). RT-PCR and Sanger sequencing were used consistently, whereas next-generation sequencing was first used in 2014, with more regular use since 2021 (appendix p 10).

**Figure 3:**
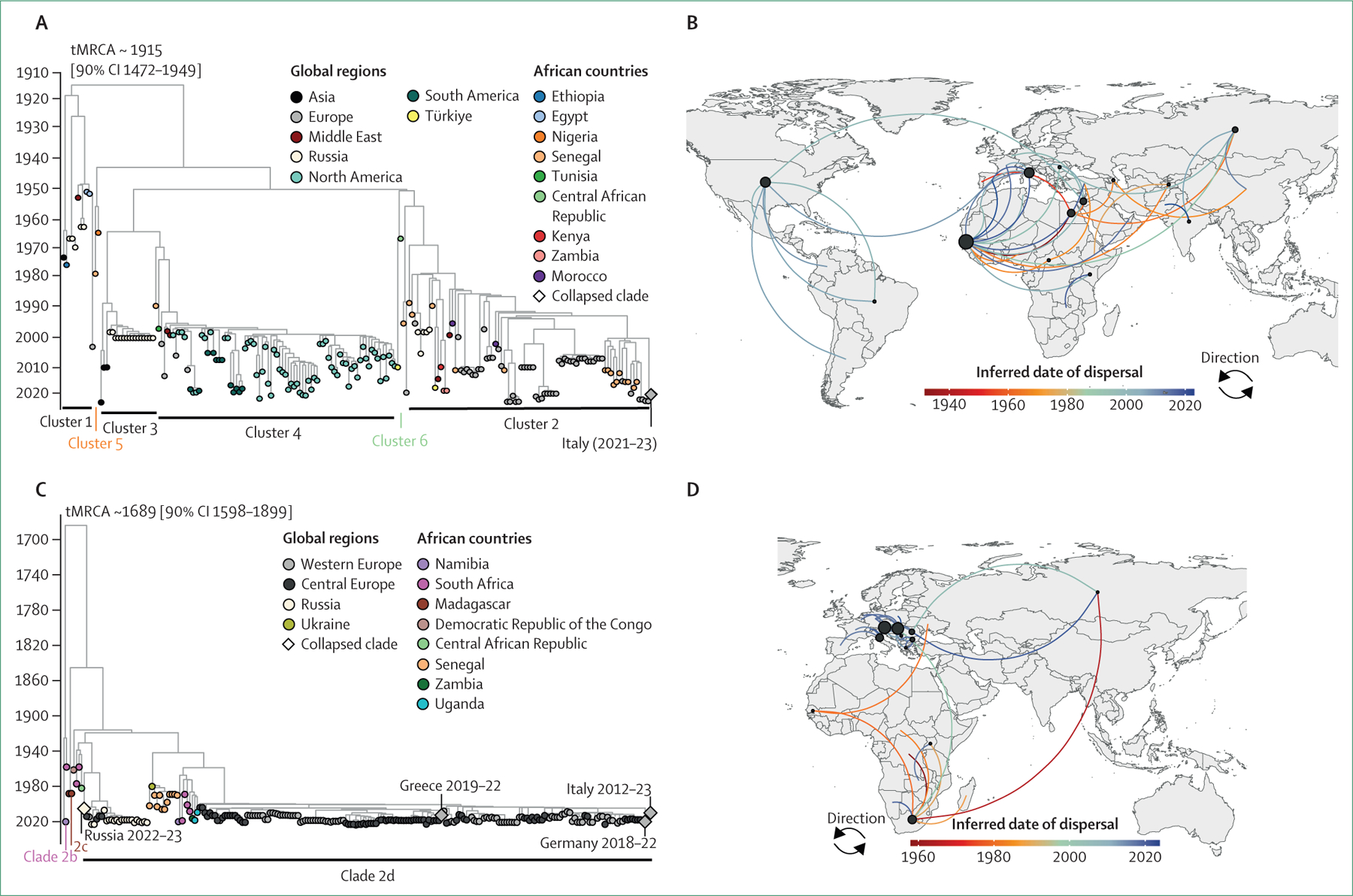
Phylogenetic reconstructions (A) Maximum likelihood time-scaled tree depicting the global diversity of West Nile virus L1A strains. The colours of the tree tips represent the global regions or African countries from which the strains were collected. Diamonds represent large clades of single origin sequences (collapsed for visualisation purposes). (B) Spatiotemporal dissemination patterns of L1A across and within Africa, Europe, Asia, and the Americas. Inferred dispersal pathways are coloured by the mean date of viral transitions for each route. Circles represent the inferred number of exports per country (the largest circle represents 20 exports, whereas the smallest circle represents one export) and are plotted in the country centre. The direction of movement is shown from the origin (black dot) to the destination, with curved lines anticlockwise in the direction of the curve. (C) Time-scaled phylogeny of L2 genomes. The colours of tree tips represent the European regions or African countries of sample collection. (D) Global geographical diffusion pattern of L2 genomes. Circles show the inferred number of exports per country (the largest circle represents 23 exports, whereas the smallest circle represents one export). L1A=lineage 1A. L2=lineage 2. tMRCA=time from the most recent common ancestor.

WNV has up to nine genetically distinct lineages: lineage 1 (L1) to 9 (L9).^65^ Globally, whole genomes are available for six of these lineages. Historically, L1A and L1B were the predominant circulating lineages, but since 2010, L2 has become more prevalent (appendix p 9). Africa has high genetic diversity, including L1A, L2, L8, and Koutango viruses, with L1A and L2 dominating (figure 3). L7 and L8 have been sequenced only from Senegal.

### Senegal is a dispersal hot spot for L1A

Our molecular clock regression analysis identified a strong correlation for L1A (clock rate=0⋅00043, r^2^=0⋅94), and indicates an almost global distribution (figure 3). The time from the most recent common ancestor (tMRCA) for taxa in the L1A tree is estimated at 1915 (90% marginal probability distribution=1472–1949). The credible interval is wide, indicating uncertainty in the estimates, which could be due to the temporal distribution of the sequence data. Such limitations of the dataset might reduce the effective temporal resolution of the analysis. However, our tMRCA dating corresponds with those reported in other studies.^11,66^ The early-diverging clade of the tree (cluster 1)^67^ consists of the oldest strains sampled from Egypt in 1951. The tree branches into larger clades (clusters 3 and 4), rooted by older African taxa from Senegal and Nigeria in cluster 5. The crown of the phylogeny depicts transmission within Europe (with the earliest clade divergence dated 1984, 90% CI 1843–1996), with strains from Senegal (2012–18) and Morocco (1996 and 2003) being basal within this cluster.

We inferred a west African origin for L1A, with the earliest estimated transition between Senegal and Egypt in 1931 (figure 3), aligning with previous findings.^11^ We observed several early dispersal events from Africa to other global regions. Within Africa, L1A strains are estimated to have dispersed from Senegal to Nigeria in 1965, Central African Republic in 1966, Morocco in 1996, Tunisia in 1997, and Kenya in 1998 and 2001. Overall, at least four dispersal events were found from Africa to Europe and Asia, with three transitions back into Africa from Europe and one from Asia. We infer three introductions from Africa into Middle Eastern countries, with seven transitions within Africa. Similar to the data reported in other studies,^11,68^ the greatest number of African exports originated from Senegal.

### The first intercontinental dispersal of L2 strains occurred from Africa to Asia

A modest clock correlation was observed for L2 (clock rate=0⋅00026, r^2^=0⋅53). The estimated tMRCA for taxa in the tree is approximately 1689 (90% CI 1598–1899), slightly earlier than the estimate by Mencattelli and colleagues,^11^ which is within our confidence intervals. Of note, the genetically divergent strains from Madagascar (1978), which form clade 2a, were dropped from the phylogeny as they did not follow the molecular clock; hence, the estimated tMRCA for all L2 strains is approximately 1456.^68^ The temporal spread of available sequences is narrow, with most sequences from large, recent, homogeneous outbreaks, which possibly contribute to the wide credible interval of our tMRCA estimate. One of the oldest sequences, collected from South Africa in 1958, forms the earliest-diverging clade 2b and groups with a strain collected from Namibia in 2020 (figure 3). Two strains from Madagascar, collected in 1988, form clade 2c, which diverged in approximately 1940 (1644–1976). Another 1958 South African strain clusters in clade 2d (approximately 1972 [1815–1987]), rooted by a Democratic Republic of the Congo sequence. Senegalese sequences (1989–2006) form the base of the western and central European transmission clade, 1971 (1835–1980).

In agreement with the data of other studies,^11,68^ our phylogeographic reconstruction inferred South Africa as the primary source of L2 transitions, with the highest estimated exports (figure 3). Early transitions within Africa are inferred from South Africa to the Democratic Republic of the Congo (1958), Senegal (1978), Central African Republic (1982), Madagascar (1987), and Uganda (1989). The first intercontinental dispersal, from Africa to Asia, is estimated to have occurred in 1964. The earliest estimated transition to Europe was from Senegal to Ukraine in 1980. One exchange is estimated between Asia and Europe. Intra-African transitions are more in number than intercontinental exports from Africa, with the latest dispersal being from Uganda to Zambia (2016) and South Africa to Namibia (2020).

## Discussion

Although WNV is a priority pathogen of international concern,^5^ our analysis identified key knowledge gaps in the true burden of disease, molecular epidemiology, and distribution of WNV in Africa. We found evidence of viral circulation in 39 of the 55 African countries. Molecular data for WNV are available from 24 of these countries, but most of them have insufficient data for all hosts and vectors. Only 63 genomic sequences from 16 African countries are available publicly. For some countries, evidence of WNV circulation is solely based on studies that used serological methods, as there are no existing molecular data. Serological cross-reactivity among flaviviruses and cocirculation of multiple antigenically related flaviviruses in Africa might have resulted in an overestimation of the true extent of WNV circulation in these countries. The use of virus neutralisation tests to confirm IgM and IgG serology is important to accurately assess the incidence of cases and seroprevalence.

Our findings show that Africa is the origin of internationally important WNV lineages. However, despite early epidemics in Uganda, Sudan, the Democratic Republic of the Congo, and Kenya (as far back as 1939),^69,70^ research efforts have largely focused on the virus’s spread in North America and Europe, with comparatively little work conducted in Africa. Here, we discuss three key challenges, which are aligned with WHO’s technical brief on research prioritisation for pandemic and epidemic intelligence^71^ and could be useful starting points to address this knowledge gap.

First, we draw attention to the need for low-cost diagnostic testing for use at the point of care. Diagnostic testing for WNV, which is often self-limiting and does not have antiviral treatment options as of now, is not prioritised in low-resource settings. This low diagnostic capacity most likely contributes to underestimation of the disease burden.^72^ For example, one study showed that WNV is an under-recognised cause of neurological and febrile illness in South Africa.40 Despite being a notifiable condition in South Africa, WNV cases are under-reported, with approximately 5–15 cases reported per year by means of passive surveillance.^13^ Such under-reporting is most likely also resulting from low clinical awareness. WNV should be considered as a cause of neurological disease with syndromic testing.

Access to affordable point-of-care diagnostics is essential for early detection and increased clinical awareness. Since rapid antigen and IgM or IgG antibody diagnostic tests are available for WNV,^73,74^ assessment of their utility in endemic, resource-constrained settings is the need of the hour. Virus neutralisation tests should then follow positive antibody diagnostics. Roll-out of appropriate rapid diagnostic tests should be coordinated and supported by the Africa Centres for Disease Control and Prevention (Africa CDC) and WHO African region. For future considerations, a review of the latest advances in arboviral diagnostics highlights isothermal nucleic acid amplification technologies as promising point-of-care diagnostic tools.^75^ However, for optimal utility in resource-constrained settings, such devices need to be capable of accurately diagnosing several different arboviruses. An improved diagnostic capacity for WNV will also facilitate the development of integrated genomic surveillance systems, a second key challenge.

This Review provides evidence for WNV detection from a broad host range, multiple vector species, and many geographical regions in Africa, in addition to diverse disease and genomic surveillance capacities across Africa. Three countries stand out as the leading generators of WNV genomes in Africa: Senegal, South Africa, and Tunisia. The successful sequencing efforts from Senegal are most likely owing to the country’s advanced arboviral disease surveillance capacity, well functioning case notification and case management systems, and high preparedness for outbreaks.^76^ South Africa also has well functioning disease surveillance and case notification systems but could be restricted by the absence of a national programme on arboviral surveillance and national surveillance programmes of animals and vectors.^76^ Tunisia’s genomic data for WNV most likely emanate from its One Health surveillance,^77^ which includes human meningitis cases, equine encephalitis cases, and passive surveillance in birds.^78^

The low surveillance for WNV in some regions could be attributed to only a few African countries identifying WNV-caused disease as a notifiable medical condition: Egypt, Morocco,^79^ Tunisia,^78^ Algeria,^80^ South Africa,^81^ Sudan, South Sudan, Sierra Leone, Cameroon, and Algeria. WNV is most likely undertested elsewhere owing to the national prioritisation of other epidemic-prone diseases.^82^ Despite its global priority status, WNV is not on the Africa CDC’s priority list for epidemic-prone diseases.^82^ Aligning such priorities and harmonising research investments is essential for effective surveillance.

The surveillance of arboviruses in the African context faces several challenges,^83^ such as insufficient training, resources, mentorship, sharing of data, and collaboration.^84^ Yopa and colleagues identified the overarching barriers to implementing One Health strategies in low-income and middle-income countries as insufficient political interest, governance, and resources.^85^ As evidenced during the SARS-CoV-2 pandemic, many African countries do not have adequate sequencing infrastructure for genomic surveillance.^86^ However, the pandemic stimulated a continent-wide increase in sequencing capacity,^86^ which should now be leveraged for other infectious diseases. Indeed, several resources in the region could be leveraged to support surveillance, including the established malaria testing and control capacity,^76^ networks that support arbovirus research and surveillance,^86^ and various capacity-building initiatives to address technical gaps.^87–89^

We recognise that establishing One Health surveillance programmes—encompassing public health surveillance, equine surveillance, xenosurveillance, and testing animal groups—is challenging, costly, and not immediately feasible for many countries. Approaches should be structured for country-specific and region-specific needs and capabilities because each region faces unique challenges. Resource-constrained countries could strategically allocate resources to equine surveillance in select high-risk regions, as such an approach has shown success for early warning of human outbreaks.^90^ Additionally, the level (community, national, or regional) at which surveillance needs to be developed should be informed by the known and modelled occurrence of the disease.^62,91^ Institutions could implement elements of the successful strategies from Senegal, South Africa, Tunisia, and from multisectoral surveillance implemented by European countries.^92^ African and European countries with successful strategies should support institutions new to One Health surveillance with crucial coordination and leadership by the Africa CDC and WHO African region.

Finally, we recognise the need for genomic data from additional geographical locations in Africa. This Review identified regions with confirmed WNV circulation but insufficient molecular surveillance. We also note the paucity of molecular sampling and sequencing for Lesotho, Eswatini, and The Gambia, which are countries nestled within the borders of countries producing the highest number of WNV genomes, and for those neighbouring them. These countries also seem to have no case reporting and seroprevalence surveys. However, risk mapping shows a high potential for WNV circulation in these areas,^62^ possibly indicating transmission that has not been detected yet.

Our phylogenetic analyses showed that Africa is a predominant source of diverse viral genotypes,^67^ with recurring spread of several genotypes out of Africa. Similarly, the results of our phylogeographic analysis indicate continual viral movements between global regions.^93^ The interconnectedness of most global regions in terms of WNV transmission is attributed to long-range migrations of wild bird hosts.^94–96^ Considering the general paucity of genomic data for WNV and the continually sustained bird migrations transporting the virus, only a small proportion of the actual viral exchanges have been detected as yet. Molecular testing for WNV in birds is restricted to five countries. Further work should be done on viral dispersal from migratory areas via bird hosts.

Our phylogeographic analysis showed that the greatest number of exports of L1A were from Senegal. Senegal and Europe are well connected via the largest land bird migration network, the Afro-Palaearctic Bird Migration System.^97^ However, viraemia is transient in birds (5–7 days),^98^ and migrations can take from two to several weeks.^99^ We postulate that infected ticks carried by trans-Saharan migrant birds could facilitate long-distance introductions,^100^ as trans-stadial transfer and onward viral transmission by an argasid tick species has been reported.^101^ The vectorial competence and role of ticks in WNV transmission remains unclear to date;^8^ research in this area could greatly advance the current understanding of WNV transmission.

## Conclusions

The burden of WNV (in addition to that of other arboviruses) remains under-recognised in Africa, with fragmented data and weak surveillance. Clinical and genomic surveillance for WNV are inadequate, hindering public health responses and obscuring the true disease burden. Addressing these gaps requires a comprehensive One Health surveillance approach; however, building such a system is complex and has many challenges. Although the ideal surveillance model for each country remains unclear, this Review offers practical starting points to begin addressing these gaps: affordable point-of-care diagnostics and targeted genomic surveillance in high-priority areas.

## Supplementary Material

1

## Figures and Tables

**Table 1:** PCR testing and genomic sequencing of WNV in humans across Africa

	Subnational location	Year of sample collection	Positive samples of total number of samples (% positive)	Study type	Sample type	Context of study population	Samples collected during an outbreak (cause of outbreak)	Reference
South Africa	Ndumo, KwaZulu-Natal	1958	1 of 1 (100%)	Research	Serum	Sequencing of stored isolates from clinical specimens	No	Burt et al (2002)^27^
South Africa	Middelburg, Mpumalanga	1964	1 of 1 (100%)	Research	Serum	Sequencing of stored isolates from clinical specimens	No	Burt et al (2002)^27^
South Africa	Johannesburg, Gauteng	1968	1 of 1 (100%)	Research	Serum	Sequencing of stored isolates from clinical specimens	No	Burt et al (2002)^27^
South Africa	Bloemfontein, Free State	1989	1 of 1 (100%)	Research	Serum	Sequencing of stored isolates from clinical specimens	No	Burt et al (2002)^27^
South Africa	Pretoria, Gauteng	1989	1 of 1 (100%)	Research	Serum	Sequencing of stored isolates from clinical specimens	No	Burt et al (2002)^27^
Namibia	Ovambo	1989	1 of 1 (100%)	Research	Serum	Sequencing of stored isolates from clinical specimens	No	Burt et al (2002)^27^
Tunisia	Sfax	1997	17 of 57 (29⋅8%)	Event-based	CSF and brain specimens	Hospitalised patients with meningitis or encephalitis	Yes (WNV)	Feki et al (2005)^28^
South Africa	Mookgophong, Northern Province	2000	1 of 1 (100%)	Research	Serum	Sequencing of stored isolates from clinical specimens	No	Burt et al (2002)^27^
South Africa	Johannesburg, Gauteng	2001	1 of 1 (100%)	Research	Serum	Sequencing of stored isolates from clinical specimens	No	Burt et al (2002)^27^
Tunisia	Sahel, Monastir	2003	18 of 113 (15⋅9%)	Event-based	Serum and CSF	Hospitalised patients with suspected WNV infection	Yes (WNV)	Riabi et al (2014)^29^
Gabon	National	2007–13	0 of 436	Indicator-based	Serum	Patients with fever who were previously PCR-negative for WNV and other arboviruses	Yes (dengue and chikungunya viruses)	Simo Tchetgna et al (2018)^30^
South Africa	Gauteng, Pretoria	2008–09	1 of 190 (0⋅5%)	Research	Serum and CSF	Patients with suspected WNV infection	No	Zaayman and Venter (2012)^31^
Kenya	National	2011–14	0 of 868	Indicator-based	Blood	Patients with fever	Yes (dengue virus)	Konongoi et al (2016)^32^
Sierra Leone	Kenema	2011–14	6 of 41 (14⋅6%)	Indicator-based	Serum	Patients with fever of unknown cause	No	Boisen et al (2015)^33^
Tunisia	Sahel, Monastir	2011–14	7 of 79 (8⋅8%)	Event-based	CSF	Patients with suspected WNV infection	Yes (WNV)	Monastiri et al (2018)^34^
Senegal	National	2012–21	20 of 7912 (0⋅3%)	Indicator-based	Blood	Patients with suspected arboviral infections	No	Ndione et al (2022)^35^
Egypt	National	2013–14	0 of 160	Research	Blood	Screening for WNV in blood donors to evaluate seroprevalence of general population	No	Youssef et al (2017)^36^
Tanzania	Dar es Salaam	2013–14	2 of 12 (16⋅7%)	Research	Plasma	Patients with fever of unknown cause	No	Williams et al (2018)^37^
Tanzania	Kilombero Valley	2014–15	1 of 842 (0⋅1%)	Indicator-based	Blood	Patients with fever of unknown cause	No	Hercik et al (2017)^38^
Sudan	Darfur	2015–16	6 of 204 (2⋅9)	Event-based for malaria	Blood	Cases of fever that tested negative	Yes	(dengue virus) Ahmed et al (2019)^39^
South Africa	Gauteng	2017	2 of 219 (0⋅9%, 95% CI 0⋅11–3⋅26)	Research	CSF	Hospitalised patients experiencing acute fever of unknown cause and neurological symptoms	No	MacIntyre et al (2023)^40^
Nigeria	Borno	2018	25 of 200 (12⋅5%)	Indicator-basesd	Serum	Patients with fever of unknown cause	No	Oderinde et al (2020)^41^
Tunisia	NA	2018	48 of 95 (50⋅5%)	Research	Urine	Cases of neuroinvasive disease with confirmed WNV serology	Yes (WNV)	Gdoura et al (2022)^42^
Tunisia	National	2018	48 of 96 (50%)	Event-based	Urine	Patients with a positive PCR test who were verified by IgM ELISA as well	Yes	(WNV) Fares et al (2021)^43^
Burkina Faso	Bobo-Dioulasso and Ouagadougou	2019–21	0 of 188	Research	Serum	Patients with fever of unknown cause	No	Tinto et al (2022)^44^
South Africa	Gauteng and Mpumalanga	2019–21	40 of 441 (9⋅07%, 95% CI 6⋅73–12⋅12)	Indicator-based	Plasma	Hospitalised patients presenting with acute fever with unknown cause (infections confirmed by serology, PCR results were negative)	No	MacIntyre et al (2023)^40^
Senegal	Bounkiling, Ziguinchor, and Tivaouane	2022	3 of 228 (1⋅3%)	Indicator-based	Plasma	Patients with non-malarial febrile illness	No	Orf et al (2024)^45^
Gabon	Libreville	NA	1 of 1 (100%)	Case study	Unknown	Case report of a single acute meningoencephalitis infection	No	Mandji Lawson et al (2009)^46^

Contextual information about the sample collection, results of testing, and study type categorisation is listed. Subnational location information is NA for studies from which the location of sample collection could not be retrieved. The 95% CIs of sample positivity provided are from the primary study. CSF=cerebrospinal fluid. NA=not available. WNV=West Nile virus.

**Table 2:** Positive molecular detections of WNV from wildlife and domestic animals in Africa

	Species name	Country	Subnational location	Sample collection year	Positive samples of total samples tested (% positive)	Study type	Context of sample collection	Reference
Birds								
African golden weaver	*Ploceus subaureus*	Kenya	Bura	2014	2 of 8	Research	Screening of general population	Nyamwaya et al (2016)^47^
African mourning dove	*Streptopelia decipiens*	Kenya	Bura	2014	1 of 3	Research	Screening of general population	Nyamwaya et al (2016)^47^
African mourning dove	*S decipiens*	Kenya	Ijara	2014	1 of 3	Research	Screening of general population	Nyamwaya et al (2016)^47^
Barn swallow	*Hirundo rustica*	Kenya	Bura	2014	2 of 5	Research	Screening of general population	Nyamwaya et al (2016)^47^
Marsh tchagra	*Antichromus minutus*	Central African Republic	NA	1972	1 of 1	Research	Stored strains collected during previous studies	Berthet et al (1997)^48^
Chickens	*Gallus gallus domesticus*	Tunisia	Sejnene	2016–17	2 of 186	Indicator-based	Screening of sentinel domestic bird flocks	Amdouni et al (2020)^49^
Chickens	*G domesticus*	Madagascar	Marofondrobok, Mitsinjo district	2012–13	1 of 95	Research	Screening of sentinel domestic bird flocks	Maquart et al (2016)^50^
Emerald-spotted wood dove	*Turtur chalcospilos*	Kenya	Ijara	2014	2 of 9	Research	Screening of general population	Nyamwaya et al (2016)^47^
Golden-breasted starling	*Lamprotornis regius*	Kenya	Ijara	2014	1 of 4	Research	Screening of general population	Nyamwaya et al (2016)^47^
Golden pipit	*Tmetothylacus tenellus*	Kenya	Bura	2014	2 of 8	Research	Screening of general population	Nyamwaya et al (2016)^47^
Greater vasa parrot	*Coracopsis vasa*	Madagascar	NA	1978	1 of 1	Research	Stored strains collected during previous studies	Berthet et al (1997)^48^
Grey-headed kingfisher	*Halcyon leucocephala*	Kenya	Bura	2014	1 of 3	Research	Screening of general population	Nyamwaya et al (2016)^47^
House sparrow	*Passer domesticus*	Kenya	Bura	2014	4 of 22	Research	Screening of general population	Nyamwaya et al (2016)^47^
Laughing dove	*Spilopelia senegalensis*	Kenya	Bura	2014	3 of 24	Research	Screening of general population	Nyamwaya et al (2016)^47^
Laughing dove	*S senegalensis*	Kenya	Hola	2014	1 of 24	Research	Screening of general population	Nyamwaya et al (2016)^47^
Lesser masked weaver	*Ploceus intermedius*	Kenya	Bura	2014	4 of 43	Research	Screening of general population	Nyamwaya et al (2016)^47^
Lesser masked weaver	*P intermedius*	Kenya	Hola	2014	10 of 43	Research	Screening of general population	Nyamwaya et al (2016)^47^
Long-billed crombec	*Sylvietta rufescens*	South Africa	KwaZulu-Natal, Ndumo	1958	1 of 1	Research	Stored strains collected during previous studies	Burt et al (2002)^27^
Namaqua dove	*Oena capensis*	Kenya	Bura	2014	4 of 24	Research	Screening of general population	Nyamwaya et al (2016)^47^
Namaqua dove	*O capensis*	Kenya	Hola	2014	1 of 24	Research	Screening of general population	Nyamwaya et al (2016)^47^
Nightjar	Caprimulgidae	Kenya	Ijara	2014	1 of 1	Research	Screening of general population	Nyamwaya et al (2016)^47^
Nubian woodpecker	*Campethera nubica*	Kenya	Ijara	2014	1 of 6	Research	Screening of general population	Nyamwaya et al (2016)^47^
Ostrich	*Struthio camelus*	South Africa	Western Cape, Prince Albert	1994	1 of 1	Research	Stored strains collected during previous studies	Burt et al (2002)^27^
Red-billed quelea	*Quelea quelea*	Kenya	Bura	2014	9 of 68	Research	Screening of general population	Nyamwaya et al (2016)^47^
Red-billed quelea	*Q quelea*	Kenya	Hola	2014	3 of 68	Research	Screening of general population	Nyamwaya et al (2016)^47^
Ring-necked dove	*Streptopelia capicola*	Kenya	Bura	2014	1 of 22	Research	Screening of general population	Nyamwaya et al (2016)^47^
Ring-necked dove	*S capicola*	Kenya	Ijara	2014	2 of 22	Research	Screening of general population	Nyamwaya et al (2016)^47^
Ruppel’s long-tailed starling	*Lamprotornis purpuroptera*	Kenya	Ijara	2014	2 of 10	Research	Screening of general population	Nyamwaya et al (2016)^47^
Pigeon	*Columba*	South Africa	Gauteng, Olifantsvlei	1968	1 of 1	Research	Stored strains collected during previous studies	Burt et al (2002)^27^
Violet-backed sunbird	*Anthreptes longuemarei*	Kenya	Bura	2014	1 of 1	Research	Screening of general population	Nyamwaya et al (2016)^47^
White-browed sparrow-weaver	*Plocepasser mahali*	Kenya	Bura	2014	1 of 22	Research	Screening of general population	Nyamwaya et al (2016)^47^
White-headed buffalo weaver	*Dinemellia dinemelli*	Kenya	Bura	2014	1 of 15	Research	Screening of general population	Nyamwaya et al (2016)^47^
White-headed buffalo weaver	*D dinemelli*	Kenya	Ijara	2014	3 of 15	Research	Screening of general population	Nyamwaya et al (2016)^47^
White-throated bee-eater	*Merops albicollis*	Kenya	Bura	2014	1 of 2	Research	Screening of general population	Nyamwaya et al (2016)^47^
Mallard duck	*Anas platyrhynchos*	Tunisia	Ouardanine Oued el Guelta	2013	1 of 2	Event-based	Screening of general population (coinciding with WNV outbreak in humans)	Monastiri et al (2018)^34^
Horses								
⋅⋅	⋅⋅	South Africa	NA	2008–15	79 of 1069 (7⋅3%)	Research	PCR testing of animals with WNV symptoms	Venter et al (2017)^51^
⋅⋅	⋅⋅	Morocco	Ouled Slama	2003	1 of 1	Event-based	PCR testing of animals with WNV symptoms (during equine outbreak)	Schuffenecker et al (2005)^52^
⋅⋅	⋅⋅	Morocco	Ameur Seflia	2003	4 of 4	Event-based	PCR testing of animals with WNV symptoms (during equine outbreak)	Schuffenecker et al (2005)^52^
⋅⋅	⋅⋅	Morocco	Mograne	2003	3 of 3	Event-based	PCR testing of animals with WNV symptoms (during equine outbreak)	Schuffenecker et al (2005)^52^
⋅⋅	⋅⋅	South Africa	Western Cape, Somerset West	1996	1 of 1	Research	Stored strains collected during previous studies	Burt et al (2002)^27^
⋅⋅	⋅⋅	South Africa	Western Cape, Ceres	2010	1 of 1	Case study	PCR testing of animals with WNV symptoms	Venter et al (2011)^53^
⋅⋅	⋅⋅	South Africa	Gauteng, Pretoria, Tiegerpoort	2008	1*	Research	PCR testing of animals with WNV symptoms	Venter et al (2009)^54^
⋅⋅	⋅⋅	South Africa	Gauteng, Midrand	2008	3*	Research	PCR testing of animals with WNV symptoms	Venter et al (2009)^54^
⋅⋅	⋅⋅	South Africa	Pretoria, Hammanskraal	2008	1*	Research	PCR testing of animals with WNV symptoms	Venter et al (2009)^54^
⋅⋅	⋅⋅	South Africa	North-Western province, Potchefstroom	2008	1*	Research	PCR testing of animals with WNV symptoms	Venter et al (2009)^54^
⋅⋅	⋅⋅	South Africa	Gauteng	2008–11	5 of 6	Research	PCR testing of animals with WNV symptoms	Williams et al (2014)^55^
⋅⋅	⋅⋅	South Africa	Western Cape	2008–11	1 of 1	Research	PCR testing of animals with WNV symptoms	Williams et al (2014)^55^
Livestock or domestic	animals							
Ayrshire cow	*Bos taurus*	South Africa	NA	2008–15	1 of 132	Research	PCR testing of animals with WNV symptoms	Venter et al (2017)^51^
Boer goat	*Capra aegagrus hircus*	South Africa	NA	2008–15	1 of 132	Research	PCR testing of animals with WNV symptoms	Venter et al (2017)^51^
Bovid	*B taurus*	South Africa	Gauteng and Free State	2010–18	2 of 93 (2⋅2%, 95% CI 0⋅0–5⋅1)	Indicator-based	PCR testing of animals with WNV symptoms or those found dead	Steyn et al (2019)^56^
Dog	*Canis lupus familiaris*	Botswana	Gaborone	1977	1 of 1	Research	Stored strains collected during previous studies	Burt et al (2002)^27^
Dog	*C familiaris*	South Africa	Gauteng	2010–18	1 of 22 (4⋅6%, 95% CI 0⋅0–13⋅3)	Indicator-based	PCR testing of animals with WNV symptoms or those found dead	Steyn et al (2019)^56^
Goat	*C hircus*	South Africa	Gauteng	2010–18	1 of 11 (9⋅1%, 95% CI 0⋅0–26⋅1)	Indicator-based	PCR testing of animals with WNV symptoms or those found dead	Steyn et al (2019)^56^
Hamster	⋅⋅	Mozambique	Mopeia	1972	1 of 1	Research	Stored strains collected during previous studies	Burt et al (2002)^27^
Sheep	*Ovis aries*	South Africa	Gauteng	2010–18	1 of 45 (2⋅2%, 95% CI 0⋅0–6⋅6)	Indicator-based	PCR testing of animals with WNV symptoms or those found dead	Steyn et al (2019)^56^
Wildlife								
African buffalo	*Syncerus caffer*	South Africa	Limpopo	2010–18	1 of 54 (1⋅9%, 95% CI 0⋅0–5⋅5)	Indicator-based	PCR testing of animals with WNV symptoms or those found dead	Steyn et al (2019)^56^
Crocodile	*Crocodylus niloticus*	Zambia	Southern Province	2019	2 of 11	Event-based	PCR testing of animals with WNV symptoms	Simulundu et al (2020)^2^
Fallow deer	*Dama dama*	South Africa	Gauteng	2010–18	1 of 3 (33⋅3%, 95% CI 0⋅0–86⋅7)	Indicator-based	PCR testing of animals with WNV symptoms or those found dead	Steyn et al (2019)^56^
Giraffe	*Giraffa giraffa*	South Africa	North West	2010–18	1 of 6 (16⋅6%, 95% CI 0⋅0–46⋅5)	Indicator-based	PCR testing of animals with WNV symptoms or those found dead	Steyn et al (2019)^56^
Lion	*Panthera leo*	South Africa	Mpumalanga	2010–18	1 of 9 (11⋅1%, 95% CI 0⋅0–31⋅2)	Indicator-based	PCR testing of animals with WNV symptoms or those found dead	Steyn et al (2019)^56^
North American white-tailed deer	*Odocoileus virginianus*	South Africa	NA	2008–15	1 of 206	Research	PCR testing of animals with WNV symptoms	Venter et al (2017)^51^
Roan antelope	*Hippotragus equinus*	South Africa	Free State and Limpopo	2010–18	2 of 28 (7⋅1%, 95% CI 0⋅0–16⋅7)	Indicator-based	PCR testing of animals with WNV symptoms or those found dead	Steyn et al (2019)^56^
Senegal bushbaby	*Galago senegalensis*	Senegal	NA	1979	1 of 1	Research	Stored strains collected during previous studies	Berthet et al (1997)^48^

Subnational location information is NA for studies from which the location of sample collection could not be retrieved. Positivity rate and 95% CIs are provided wherever available from the primary study. The percentage positivity is not provided for studies that did not use a sampling method representative of the populations. The study type and context of sample collection are provided. *The total sample per location is not reported in the original study. NA=not available. WNV=West Nile virus.

## References

[1] Petersen LR, Brault AC, Nasci RS. West Nile virus: review of the literature. JAMA 2013; 310: 308–15. 23860989 10.1001/jama.2013.8042 PMC4563989

[2] Simulundu E, Ndashe K, Chambaro HM, West Nile virus in farmed crocodiles, Zambia, 2019. Emerg Infect Dis 2020; 26: 811–14. 32187004 10.3201/eid2604.190954 PMC7101096

[3] Smithburn KC, Hughes TP, Burke AW, Paul JH. A neurotropic virus isolated from the blood of a native of Uganda. Am J Trop Med Hyg 1940; s1–20: 471–92.

[4] MacDonald RD, Krym VF. West Nile virus. Primer for family physicians. Can Fam Physician 2005; 51: 833–37. 15986939 PMC1479528

[5] WHO. Pathogens prioritization: a scientific framework for epidemic and pandemic research preparedness. July 30, 2024. https://www.who.int/publications/m/item/pathogens-prioritization-a-scientific-framework-for-epidemic-and-pandemic-research-preparedness (accessed Nov 27, 2024).

[6] US Centers for Disease Control and Prevention. West Nile virus https://www.cdc.gov/west-nile-virus/data-maps/historic-data.html (accessed Nov 27, 2024).

[7] European Centre for Disease Prevention and Control. Epidemiological update: West Nile virus transmission season in Europe, 2023. Feb 20, 2024. https://www.ecdc.europa.eu/en/news-events/epidemiological-update-west-nile-virus-transmission-season-europe-2023-0 (accessed Sept 16, 2024).

[8] Mencattelli G, Ndione MHD, Rosà R, Epidemiology of West Nile virus in Africa: an underestimated threat. PLoS Negl Trop Dis 2022; 16: e0010075. 35007285 10.1371/journal.pntd.0010075 PMC8789169

[9] Dellicour S, Lequime S, Vrancken B, Epidemiological hypothesis testing using a phylogeographic and phylodynamic framework. Nat Commun 2020; 11: 5620. 33159066 10.1038/s41467-020-19122-z PMC7648063

[10] Aguilera-Sepúlveda P, Cano-Gómez C, Villalba R, The key role of Spain in the traffic of West Nile virus lineage 1 strains between Europe and Africa. Infect Dis (Lond) 2024; 56: 743–58. 38836293 10.1080/23744235.2024.2348633

[11] Mencattelli G, Ndione MHD, Silverj A, Spatial and temporal dynamics of West Nile virus between Africa and Europe. Nat Commun 2023; 14: 6440. 37833275 10.1038/s41467-023-42185-7 PMC10575862

[12] Hadfield J, Brito AF, Swetnam DM, Twenty years of West Nile virus spread and evolution in the Americas visualized by Nextstrain. PLoS Pathog 2019; 15: e1008042. 31671157 10.1371/journal.ppat.1008042 PMC6822705

[13] Sule WF, Oluwayelu DO, Hernández-Triana LM, Fooks AR, Venter M, Johnson N. Epidemiology and ecology of West Nile virus in sub-Saharan Africa. Parasit Vectors 2018; 11: 414. 30005653 10.1186/s13071-018-2998-y PMC6043977

[14] Lemey P, Rambaut A, Drummond AJ, Suchard MA. Bayesian phylogeography finds its roots. PLoS Comput Biol 2009; 5: e1000520. 19779555 10.1371/journal.pcbi.1000520 PMC2740835

[15] Minin VN, Bloomquist EW, Suchard MA. Smooth skyride through a rough skyline: Bayesian coalescent-based inference of population dynamics. Mol Biol Evol 2008; 25: 1459–71. 18408232 10.1093/molbev/msn090 PMC3302198

[16] Stockdale JE, Liu P, Colijn C. The potential of genomics for infectious disease forecasting. Nat Microbiol 2022; 7: 1736–43. 36266338 10.1038/s41564-022-01233-6

[17] Ladner JT, Grubaugh ND, Pybus OG, Andersen KG. Precision epidemiology for infectious disease control. Nat Med 2019; 25: 206–11. 30728537 10.1038/s41591-019-0345-2 PMC7095960

[18] Liberati A, Altman DG, Tetzlaff J, The PRISMA statement for reporting systematic reviews and meta-analyses of studies that evaluate health care interventions: explanation and elaboration. PLoS Med 2009; 6: e1000100. 19621070 10.1371/journal.pmed.1000100 PMC2707010

[19] Vansteelant WMG, Gangoso L, Bouten W, Viana DS, Figuerola J. Adaptive drift and barrier-avoidance by a fly-forage migrant along a climate-driven flyway. Mov Ecol 2021; 9: 37. 34253264 10.1186/s40462-021-00272-8 PMC8276455

[20] Trevail AM, Nicoll MAC, Freeman R, Tracking seabird migration in the tropical Indian Ocean reveals basin-scale conservation need. Curr Biol 2023; 33: 5247–56.e4. 37972589 10.1016/j.cub.2023.10.060

[21] WHO. Integrated disease surveillance and response technical guidelines: booklet two: sections 1, 2, and 3, 3rd ed. Jan 1, 2019. https://www.who.int/publications/i/item/WHO-AF-WHE-CPI-01-2019 (accessed Aug 26, 2024).

[22] Crowe S, Cresswell K, Robertson A, Huby G, Avery A, Sheikh A. The case study approach. BMC Med Res Methodol 2011; 11: 100. 21707982 10.1186/1471-2288-11-100 PMC3141799

[23] Berger S West Nile fever: global status. GIDEON Informatics, 2023.

[24] Minh BQ, Schmidt HA, Chernomor O, IQ-TREE 2: new models and efficient methods for phylogenetic inference in the genomic era. Mol Biol Evol 2020; 37: 1530–34. 32011700 10.1093/molbev/msaa015 PMC7182206

[25] Sagulenko P, Puller V, Neher RA. TreeTime: maximum-likelihood phylodynamic analysis. Virus Evol 2018; 4: vex042. 29340210 10.1093/ve/vex042 PMC5758920

[26] Mathiot CC, Georges AJ, Deubel V. Comparative analysis of West Nile virus strains isolated from human and animal hosts using monoclonal antibodies and cDNA restriction digest profiles. Res Virol 1990; 141: 533–43. 1703658 10.1016/0923-2516(90)90085-w

[27] Burt FJ, Grobbelaar AA, Leman PA, Anthony FS, Gibson GVF, Swanepoel R. Phylogenetic relationships of southern African West Nile virus isolates. Emerg Infect Dis 2002; 8: 820–26. 12141968 10.3201/eid0808.020027 PMC2732512

[28] Feki I, Marrakchi C, Ben Hmida M, Epidemic West Nile virus encephalitis in Tunisia. Neuroepidemiology 2005; 24: 1–7. 15459502 10.1159/000081042

[29] Riabi S, Gaaloul I, Mastouri M, Hassine M, Aouni M. An outbreak of West Nile virus infection in the region of Monastir, Tunisia, 2003. Pathog Glob Health 2014; 108: 148–57. 10.1179/2047773214Y.0000000137 PMC4083177 24766339

[30] Simo Tchetgna H, Sem Ouilibona R, Nkili-Meyong AA, Viral exploration of negative acute febrile cases observed during chikungunya outbreaks in Gabon. Intervirology 2018; 61: 174–84. 30625488 10.1159/000495136

[31] Zaayman D, Venter M. West Nile virus neurologic disease in humans, South Africa, September 2008-May 2009. Emerg Infect Dis 2012; 18: 2051–54. 23171668 10.3201/eid1812.111208 PMC3557887

[32] Konongoi L, Ofula V, Nyunja A, Detection of dengue virus serotypes 1, 2 and 3 in selected regions of Kenya: 2011–2014. Virol J 2016; 13: 182. 27814732 10.1186/s12985-016-0641-0 PMC5097412

[33] Boisen ML, Schieffelin JS, Goba A, Multiple circulating infections can mimic the early stages of viral hemorrhagic fevers and possible human exposure to filoviruses in Sierra Leone prior to the 2014 outbreak. Viral Immunol 2015; 28: 19–31. 25531344 10.1089/vim.2014.0108 PMC4287116

[34] Monastiri A, Mechri B, Vázquez-González A, A four-year survey (2011–2014) of West Nile virus infection in humans, mosquitoes and birds, including the 2012 meningoencephalitis outbreak in Tunisia. Emerg Microbes Infect 2018; 7: 28. 29535295 10.1038/s41426-018-0028-y PMC5849722

[35] Ndione MHD, Ndiaye EH, Faye M, Re-introduction of West Nile virus lineage 1 in Senegal from Europe and subsequent circulation in human and mosquito populations between 2012 and 2021. Viruses 2022; 14: 2720. 36560724 10.3390/v14122720 PMC9785585

[36] Youssef SR, Eissa DG, Abo-Shady RA, Seroprevalence of anti-WNV IgG antibodies and WNV-RNA in Egyptian blood donors. J Med Virol 2017; 89: 1323–29. 27603170 10.1002/jmv.24682

[37] Williams SH, Cordey S, Bhuva N, Investigation of the plasma virome from cases of unexplained febrile illness in Tanzania from 2013 to 2014: a comparative analysis between unbiased and VirCapSeq-VERT high-throughput sequencing approaches. mSphere 2018; 3: e00311–18. 30135221 10.1128/mSphere.00311-18 PMC6106054

[38] Hercik C, Cosmas L, Mogeni OD, A diagnostic and epidemiologic investigation of acute febrile illness (AFI) in Kilombero, Tanzania. PLoS One 2017; 12: e0189712. 29287070 10.1371/journal.pone.0189712 PMC5747442

[39] Ahmed A, Elduma A, Magboul B, Higazi T, Ali Y. The first outbreak of dengue fever in Greater Darfur, Western Sudan. Trop Med Infect Dis 2019; 4: 43. 30823624 10.3390/tropicalmed4010043 PMC6473713

[40] MacIntyre C, Lourens C, Mendes A, West Nile virus, an underdiagnosed cause of acute fever of unknown origin and neurological disease among hospitalized patients in South Africa. Viruses 2023; 15: 2207. 38005884 10.3390/v15112207 PMC10674603

[41] Oderinde BS, Mora-Cárdenas E, Carletti T, Baba MM, Marcello A. Prevalence of locally undetected acute infections of flaviviruses in North-Eastern Nigeria. Virus Res 2020; 286: 198060. 32561377 10.1016/j.virusres.2020.198060

[42] Gdoura M, Fares W, Bougatef S, The value of West Nile virus RNA detection by real-time RT-PCR in urine samples from patients with neuroinvasive forms. Arch Microbiol 2022; 204: 238. 35366683 10.1007/s00203-022-02829-6

[43] Fares W, Gdoura M, Dhrif H, Genetic characterization of West Nile virus strains during neuroinvasives infection outbreak in Tunisia, 2018. Transbound Emerg Dis 2021; 68: 2414–21. 33128297 10.1111/tbed.13905

[44] Tinto B, Kaboré DPA, Kagoné TS, Screening of circulation of Usutu and West Nile viruses: a One Health approach in humans, domestic animals and mosquitoes in Burkina Faso, West Africa. Microorganisms 2022; 10: 2016. 36296292 10.3390/microorganisms10102016 PMC9610586

[45] Orf GS, Ahouidi AD, Mata M, Next-generation sequencing survey of acute febrile illness in Senegal (2020–2022). Front Microbiol 2024; 15: 1362714. 38655084 10.3389/fmicb.2024.1362714 PMC11037400

[46] Mandji Lawson JM, Mounguengui D, Ondounda M, Nguema Edzang B, Vandji J, Tchoua R. A case of meningo-encephalitis due to West Nile virus in Libreville, Gabon. Med Trop (Mars) 2009; 69: 501–02 (in French). 20025184

[47] Nyamwaya D, Wang’ondu V, Amimo J, Detection of West Nile virus in wild birds in Tana River and Garissa counties, Kenya. BMC Infect Dis 2016; 16: 696. 27881079 10.1186/s12879-016-2019-8 PMC5121970

[48] Berthet FX, Zeller HG, Drouet MT, Rauzier J, Digoutte JP, Deubel V. Extensive nucleotide changes and deletions within the envelope glycoprotein gene of Euro-African West Nile viruses. J Gen Virol 1997; 78: 2293–97. 9292017 10.1099/0022-1317-78-9-2293

[49] Amdouni J, Monaco F, Portanti O, Detection of enzootic circulation of a new strain of West Nile virus lineage 1 in sentinel chickens in the north of Tunisia. Acta Trop 2020; 202: 105223. 31647898 10.1016/j.actatropica.2019.105223

[50] Maquart M, Boyer S, Rakotoharinome VM, High prevalence of West Nile virus in domestic birds and detection in 2 new mosquito species in Madagascar. PLoS One 2016; 11: e0147589. 26807720 10.1371/journal.pone.0147589 PMC4725773

[51] Venter M, Pretorius M, Fuller JA, West Nile virus lineage 2 in horses and other animals with neurologic disease, South Africa, 2008–2015. Emerg Infect Dis 2017; 23: 2060–64. 29148375 10.3201/eid2312.162078 PMC5708237

[52] Schuffenecker I, Peyrefitte CN, el Harrak M, Murri S, Leblond A, Zeller HG. West Nile virus in Morocco, 2003. Emerg Infect Dis 2005; 11: 306–09. 15752452 10.3201/eid1102.040817 PMC3320441

[53] Venter M, Human S, van Niekerk S, Williams J, van Eeden C, Freeman F. Fatal neurologic disease and abortion in mare infected with lineage 1 West Nile virus, South Africa. Emerg Infect Dis 2011; 17: 1534–36. 21801644 10.3201/eid1708.101794 PMC3381566

[54] Venter M, Human S, Zaayman D, Lineage 2 West Nile virus as cause of fatal neurologic disease in horses, South Africa. Emerg Infect Dis 2009; 15: 877–84. 19523285 10.3201/eid1506.081515 PMC2727306

[55] Williams JH, van Niekerk S, Human S, van Wilpe E, Venter M. Pathology of fatal lineage 1 and 2 West Nile virus infections in horses in South Africa. J S Afr Vet Assoc 2014; 85: 1105. 25686260 10.4102/jsava.v85i1.1105

[56] Steyn J, Botha E, Stivaktas VI, West Nile virus in wildlife and nonequine domestic animals, South Africa, 2010–2018. Emerg Infect Dis 2019; 25: 2290–94. 31742510 10.3201/eid2512.190572 PMC6874268

[57] Triki H, Murri S, Guenno BL, Méningoencéphalite à arbovirus West Nile en Tunisie. Med Trop (Mars) 2001; 61: 487–90. 11980397

[58] WHO. Infectious disease outbreaks reported in the Eastern Mediterranean Region in 2018. Jan 20, 2019. https://www.emro.who.int/pandemic-epidemic-diseases/news/infectious-disease-outbreaks-reported-in-the-eastern-mediterranean-region-in-2018.html (accessed Sept 11, 2024).

[59] Roy M, Mufunda F, Slawski D, West Nile virus in crocodiles and mosquitoes in Zimbabwe. bioRxiv 2021; published online Feb 19. 10.1101/2021.02.19.431612 (preprint).

[60] Altmann M, Nahapetyan K, Asghar H. Identifying hotspots of viral haemorrhagic fevers in the eastern Mediterranean Region: perspectives for the Emerging and Dangerous Pathogens Laboratory Network. East Mediterr Health J 2019; 24: 1049–57. 30701519 10.26719/emhj.18.002

[61] Larrieu S, Cardinale E, Ocquidant P, A fatal neuroinvasive West Nile virus infection in a traveler returning from Madagascar: clinical, epidemiological and veterinary investigations. Am J Trop Med Hyg 2013; 89: 211–13. 23751400 10.4269/ajtmh.12-0003 PMC3741238

[62] García-Carrasco J-M, Muñoz A-R, Olivero J, Segura M, Real R. Mapping the risk for West Nile virus transmission, Africa. Emerg Infect Dis 2022; 28: 777–85. 35318911 10.3201/eid2804.211103 PMC8962882

[63] International BirdLife. State of Africa’s birds: indicators for our changing environment. March 5, 2018. https://www.birdlife.org/wp-content/uploads/2021/03/soab_2017-english_final.pdf (accessed Oct 28, 2024).

[64] Kontur. Worldwide population density map. https://www.kontur.io/datasets/population-dataset/ (accessed May 13, 2025).

[65] Hall RA, Scherret JH, Mackenzie JS. Kunjin virus: an Australian variant of West Nile? Ann N Y Acad Sci 2001; 951: 153–60. 11797773

[66] Maroco D, Parreira R, Dos Santos FA, Tracking the pathways of West Nile virus: phylogenetic and phylogeographic analysis of a 2024 isolate from Portugal. Microorganisms 2025; 13: 585. 40142478 10.3390/microorganisms13030585 PMC11945232

[67] May FJ, Davis CT, Tesh RB, Barrett ADT. Phylogeography of West Nile virus: from the cradle of evolution in Africa to Eurasia, Australia, and the Americas. J Virol 2011; 85: 2964–74. 21159871 10.1128/JVI.01963-10 PMC3067944

[68] Grubaugh Lab. Global West Nile virus – Nextstrain build. 2023. https://nextstrain.org/community/grubaughlab/WNV-Global (accessed April 16, 2025).

[69] Chancey C, Grinev A, Volkova E, Rios M. The global ecology and epidemiology of West Nile virus. BioMed Res Int 2015; 2015: 376230. 25866777 10.1155/2015/376230 PMC4383390

[70] Smithburn KC, Jacobs HR. Neutralization-tests against neurotropic viruses with sera collected in Central Africa. J Immunol 1942; 44: 9–23.

[71] WHO. Research prioritization for pandemic and epidemic intelligence: technical brief. May 23, 2024. https://www.who.int/publications/i/item/9789240094529 (accessed Dec 11, 2024).

[72] Hungwe FT, Laycock KM, Ntereke TD, Mabaka R, Paganotti GM. A historical perspective on arboviruses of public health interest in Southern Africa. Pathog Glob Health 2024; 118: 131–59. 38082563 10.1080/20477724.2023.2290375 PMC11141323

[73] Biopanda Reagents Ltd. West Nile virus IgG/IgM rapid test. https://www.biopanda.co.uk/products/clinical-rapid-tests/infectious-disease/west-nile-virus-iggigm-rapid-test (accessed April 16, 2025).

[74] Panella NA, Burkhalter KL, Langevin SA, Rapid West Nile virus antigen detection. Emerg Infect Dis 2005; 11: 1633–35. 16318713 10.3201/eid1110.040394 PMC3366728

[75] Varghese J, De Silva I, Millar DS. Latest advances in arbovirus diagnostics. Microorganisms 2023; 11: 1159. 37317133 10.3390/microorganisms11051159 PMC10223626

[76] WHO. Surveillance and control of arboviral diseases in the WHO African region: assessment of country capacities. Nov 29, 2022. https://www.who.int/publications/i/item/9789240052918 (accessed Jan 19, 2024).

[77] Dente MG, Riccardo F, Bolici F, Implementation of the One Health approach to fight arbovirus infections in the Mediterranean and Black Sea region: assessing integrated surveillance in Serbia, Tunisia and Georgia. Zoonoses Public Health 2019; 66: 276–87. 30724030 10.1111/zph.12562 PMC6850493

[78] Hammami S, Hassine TB, Conte A, West Nile disease in Tunisia: an overview of 60 years. Vet Ital 2017; 53: 225–34. 29152704 10.12834/VetIt.1181.6565.2

[79] García-Carrasco J, Muñoz A, Olivero J, Segura M, Real R. An African West Nile virus risk map for travellers and clinicians. Travel Med Infect Dis 2023; 52: 102529. 36549415 10.1016/j.tmaid.2022.102529

[80] Lafri I, Hachid A, Bitam I. West Nile virus in Algeria: a comprehensive overview. New Microbes New Infect 2018; 27: 09–13. 10.1016/j.nmni.2018.10.002 PMC6260397 30519477

[81] National Institute for Communicable Diseases. South Africa. Notifiable medical conditions resources. https://www.nicd.ac.za/nmc-overview/nmc-resources/ (accessed May 13, 2025).

[82] Africa Centers for Disease Control and Prevention. Risk ranking and prioritization of epidemic-prone diseases. Feb 26, 2023. https://africacdc.org/download/risk-ranking-and-prioritization-of-epidemic-prone-diseases/ (accessed Oct 30, 2024).

[83] Agboli E, Tomazatos A, Maiga-Ascofaré O, Arbovirus epidemiology: the mystery of unnoticed epidemics in Ghana, West Africa. Microorganisms 2022; 10: 1914. 36296190 10.3390/microorganisms10101914 PMC9610185

[84] Braack L, Wulandhari SA, Chanda E, Developing African arbovirus networks and capacity strengthening in arbovirus surveillance and response: findings from a virtual workshop. Parasit Vectors 2023; 16: 129. 37059998 10.1186/s13071-023-05748-7 PMC10103543

[85] Yopa DS, Massom DM, Kiki GM, Barriers and enablers to the implementation of One Health strategies in developing countries: a systematic review. Front Public Health 2023; 11: 1252428. 38074697 10.3389/fpubh.2023.1252428 PMC10701386

[86] Tegally H, San JE, Cotten M, The evolving SARS-CoV-2 epidemic in Africa: insights from rapidly expanding genomic surveillance. Science 2022; 378: eabq5358. 36108049 10.1126/science.abq5358 PMC9529057

[87] Dadzie SK, Akorli J, Coulibaly MB, Building the capacity of West African countries in Aedes surveillance: inaugural meeting of the West African Aedes Surveillance Network (WAASuN). Parasit Vectors 2022; 15: 381. 36271451 10.1186/s13071-022-05507-0 PMC9585720

[88] Obame-Nkoghe J, Agossou AE, Mboowa G, Climate-influenced vector-borne diseases in Africa: a call to empower the next generation of African researchers for sustainable solutions. Infect Dis Pover 2024; 13: 26. 10.1186/s40249-024-01193-5 PMC10938833 38486340

[89] CLIMADE. Training and capacity building. 2022. https://climade.health/training-and-capacity-building/ (accessed Nov 1, 2024).

[90] Costa EA, Bayeux JJ, Silva AS, Epidemiological surveillance of West Nile virus in the world and Brazil. Braz J Vet Res Anim Sci 2020; 56: e164335.

[91] Conley AK, Fuller DO, Haddad N, Hassan AN, Gad AM, Beier JC. Modeling the distribution of the West Nile and Rift Valley Fever vector Culex pipiens in arid and semi-arid regions of the Middle East and North Africa. Parasit Vectors 2014; 7: 289. 24962735 10.1186/1756-3305-7-289 PMC4077837

[92] Tegegne HA, Freeth FTA, Bogaardt C, Implementation of One Health surveillance systems: opportunities and challenges - lessons learned from the OH-EpiCap application. One Health 2024; 18: 100704. 38496337 10.1016/j.onehlt.2024.100704 PMC10940803

[93] McMullen AR, Albayrak H, May FJ, Davis CT, Beasley DWC, Barrett ADT. Molecular evolution of lineage 2 West Nile virus. J Gen Virol 2013; 94: 318–25. 23136360 10.1099/vir.0.046888-0 PMC3709619

[94] Parreira R, Severino P, Freitas F, Piedade J, Almeida APG, Esteves A. Two distinct introductions of the West Nile virus in Portugal disclosed by phylogenetic analysis of genomic sequences. Vector Borne Zoonotic Dis 2007; 7: 344–52. 17896871 10.1089/vbz.2006.0632

[95] Mancuso E, Cecere JG, Iapaolo F, West Nile and Usutu virus introduction via migratory birds: a retrospective analysis in Italy. Viruses 2022; 14: 416. 35216009 10.3390/v14020416 PMC8880244

[96] García-Carrasco JM, Muñoz AR, Olivero J, Figuerola J, Fa JE, Real R. Gone (and spread) with the birds: can chorotype analysis highlight the spread of West Nile virus within the Afro-Palaearctic flyway? One Health 2023; 17: 100585. 37359749 10.1016/j.onehlt.2023.100585 PMC10285635

[97] Hahn S, Bauer S, Liechti F. The natural link between Europe and Africa – 2.1 billion birds on migration. Oikos 2009; 118: 624–26.

[98] Komar N, Langevin S, Hinten S, Experimental infection of North American birds with the New York 1999 strain of West Nile virus. Emerg Infect Dis 2003; 9: 311–22. 12643825 10.3201/eid0903.020628 PMC2958552

[99] Zwarts L, Bijlsma RG, van der Kamp J. Seasonal shifts in habitat choice of birds in the Sahel and the importance of ‘refuge trees’ for surviving the dry season. Ardea 2023; 111: 227–50.

[100] Mancuso E, Toma L, Pascucci I, Direct and indirect role of migratory birds in spreading CCHFV and WNV: a multidisciplinary study on three stop-over islands in Italy. Pathogens 2022; 11: 1056. 36145488 10.3390/pathogens11091056 PMC9505975

[101] Lawrie CH, Uzcátegui NY, Gould EA, Nuttall PA. Ixodid and argasid tick species and West Nile virus. Emerg Infect Dis 2004; 10: 653–57. 15200855 10.3201/eid1004.030517 PMC3323096

