# Supplementary material for: Identifying genomic surveillance gaps in Africa for the global public health response to West Nile virus: a systematic review
Source: Lancet Microbe. Author manuscript; Available in PMC 2026 Jan 6. (PMC12769835; doi:10.1016/j.lanmic.2025.101176)
Supplement: 1 [file NIHMS2116984-supplement-1.pdf]

THE LANCET
Microbe

Supplementary appendix

This appendix formed part of the original submission and has been peer reviewed.
We post it as supplied by the authors.

Supplement to: Moir M, Sitharam N, Hofstra LM, et al. Identifying genomic surveillance gaps in Africa for the global public health response to West Nile virus: a systematic review. *Lancet Microbe* 2025. https://doi.org/10.1016/j.lanmic.2025.101176

Identifying genomic surveillance gaps in Africa for the global public health response to West Nile Virus: a systematic review

Appendix table 1. Table of search terms employed in the literature search.

Search	Terms
#1	"West Nile virus"[Mesh] OR "West Nile Fever"[Mesh] OR “West nile virus”[tiab] OR WNV[tiab] OR “West nile fever”[tiab] OR “West nile encephalitis”[tiab] OR “West nile meningitis”[tiab] OR “West nile poliomyelitis”[tiab]<br>37,502 search results
#2	Africa[tiab] OR Algeria[tiab] OR Angola[tiab] OR Benin[tiab] OR Botswana[tiab] OR "Burkina Faso"[tiab] OR Burundi[tiab] OR "Cabo Verde"[tiab] OR Cameroon[tiab] OR "Canary Islands"[tiab] OR "Cape Verde"[tiab] OR "Central African Republic"[tiab] OR Chad[tiab] OR Comoros[tiab] OR Congo[tiab] OR "Democratic Republic of Congo"[tiab] OR DRC[tiab] OR Djibouti[tiab] OR Egypt[tiab] OR "Equatorial Guinea"[tiab] OR Eritrea[tiab] OR Eswatini[tiab] OR Ethiopia[tiab] OR Gabon[tiab] OR Gambia[tiab] OR Ghana[tiab] OR Guinea[tiab] OR "Guinea Bissau"[tiab] OR "Ivory Coast"[tiab] OR "Cote d'Ivoire"[tiab] OR Jamahiriya[tiab] OR Kenya[tiab] OR Lesotho[tiab] OR Liberia[tiab] OR Libya[tiab] OR Madagascar[tiab] OR Malawi[tiab] OR Mali[tiab] OR Mauritania[tiab] OR Mauritius[tiab] OR Mayotte[tiab] OR Morocco[tiab] OR Mozambique[tiab] OR Namibia[tiab] OR Niger OR Nigeria OR Principe OR Reunion OR Rwanda OR "Sao Tome" OR Senegal[tiab] OR Seychelles[tiab] OR "Sierra Leone"[tiab] OR Somalia[tiab] OR "South Africa"[tiab] OR “Southern Provinces”[tiab] OR "St Helena"[tiab] OR Sudan[tiab] OR Swaziland[tiab] OR Tanzania[tiab] OR Togo[tiab] OR Tunisia[tiab] OR Uganda[tiab] OR "Western Sahara"[tiab] OR Zaire[tiab] OR Zambia[tiab] OR Zimbabwe[tiab]<br>1,254,281 search results
#3	"Genetic Variation" [Mesh] OR "Genome, Viral" [Mesh] OR Genomics [Mesh] OR Phylogeny [Mesh] OR Metagenome [Mesh] OR Lineage [tiab] OR Strain [tiab] OR Genotype [tiab] OR "Molecular epidemiology" [Mesh] OR "Nucleic acids" [Mesh] OR Genetic [tiab] OR Phylogenetic* [tiab] OR Phylogeo* [tiab] OR Gene [tiab]<br>4,538,579 search results
#4	NOT Review [publication type]

Appendix figure 1. Publication identification, screening, and inclusion results for literature review according to PRISMA criteria.

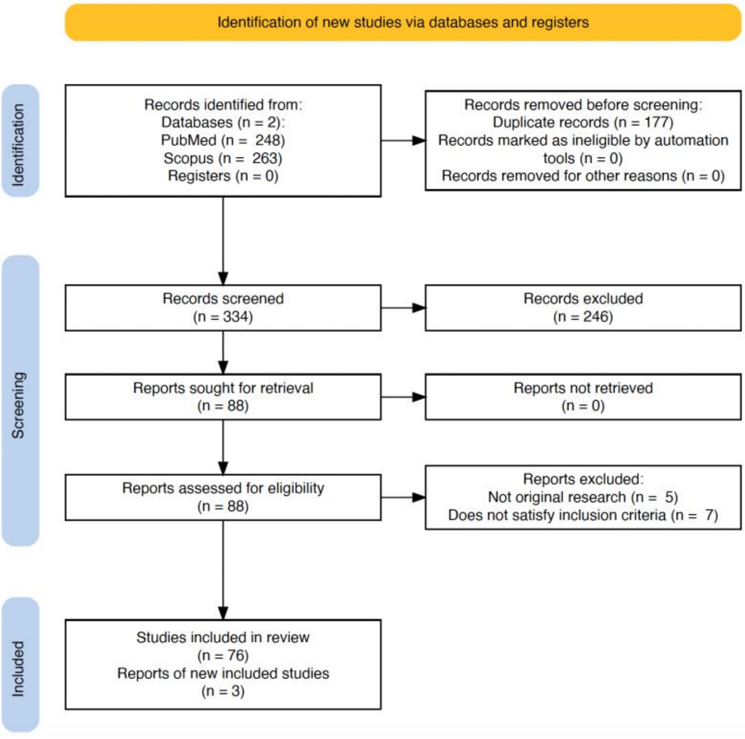

Appendix figure 2. Large treemap depicts the number of West Nile virus sequences retrieved from the literature review (n=258) of which 26 are unpublished. The smaller treemap shows the composition of accessioned sequences that were not captured by the literature review (n=58) and that 40 sequences are not linked to publications. Table lists the accession numbers in each of these categories.

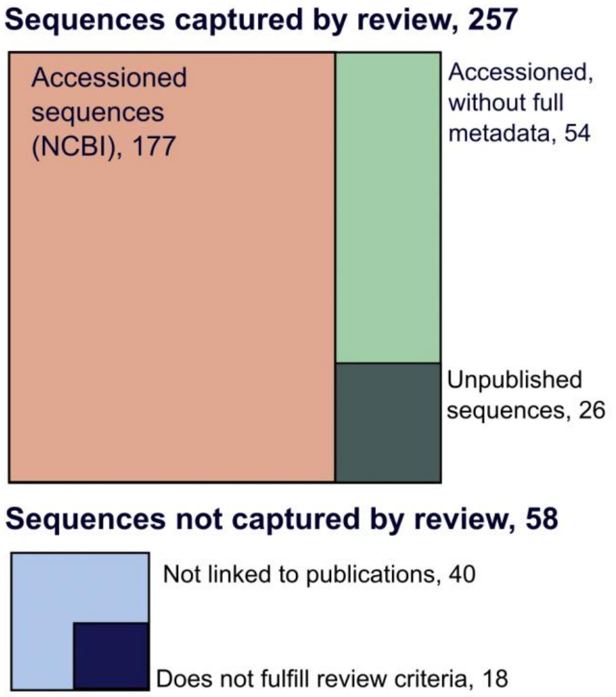

Captured by review	
Accessioned sequences	AF146082,AY701412,AY701413,EF429197,EF429198,EF429199,EF429200,FJ464377, FJ464378,FJ464379,FJ464380,FJ464381,GQ851606,GQ851607,GQ851608,HQ594469, HQ594470,JN393308,JX974605,KC243146,KP099553,KP099554,KP099555,KP099556, KP099557,KP099558,KP099559,KP099560,KP099561,KX181348,KX181349,KX181350, KX181351,KX181352,KX181353,KX181354,KX189174,KX189175,KX189176,KY176717, KY176718,KY176719,KY176720,KY176721,KY176722,KY176723,KY176724,KY176725, KY176726,KY176727,KY176728,KY176729,KY176730,KY176731,KY176732,KY176733, KY176734,KY176735,KY176736,KY703854,KY703855,KY703856,LC318700,LC489409, LC518897,MF371349,MF371350,MF371351,MF371352,MF371353,MF371354,MF371355, MF371356,MF371357,MF371358,MF371359,MF371360,MN270988,MN270989,MN270990, MT055864,MT055865,MT055866,MT055867,MT055868,MT055869,MT055870,MT055871, MT055872,MT055873,MT055874,MT055875,MT055876,MT055877,MT055878,MT055879, MT055880,MT055881,MT055882,MT055883,MT055884,MT055885,MT055886,MT132377, MT132378,MT132379,MT132380,MT132381,MT132382,MT132383,MT132384,MT132385, MT132386,MT132387,MT132388,MT132389,MW383507,MW383508,MW383509,MW436414, MW436415,MW436416,MW436417,MW436418,MW436419,MW436420,OL411950,OL411951, OL411952,OL411953,OL411954,OL411955,OL411956,OL411957,OL411958,OL411959, OL411960,OL411963,OL411964,OL411965,OL411966,OL411967,OL790166,OL790167, OM728607,ON813210,ON813211,ON813212,ON813213,ON813214,ON813215,ON813216, ON813217,ON813218,ON813219,FJ464376,OP846971,OP846972,OP846973,OP846974, OP846975,OP846976,OP846977,OP846978,OP846979,OP846980,OP846981,OP846982, OP870453,OP870454,OP870455,OP870456,OP870457,OP870458,OP870459,OP870460, OP870461
Accessioned without full metadata	AY268133,AY262283,AF001556-AF001574,MN057643,PP445046,AF514918-AF514946, AF260968,M12294

Not captured by review	
Not linked to publicat	FJ464382,DQ055173,DQ318019,DQ318020,EU081844,HQ594467,HQ594468,JN226820, JN226821,JN226822,JN226823,JN226824,JN226825,JN226826,JN226827,JN226828, JN226829,JN226830,JN226831,JN226832,KM052152,KU978767,KY523178,OL790150, OL790151,OL790152,OL790153,OL790154,OL790155,OL790156,OL790157,OL790158, OL790159,OL790160,OL790161,OL790162,OL790163,OL790164,OL790165,OP345226
Does not fulfil review criteria	AF205880,AF205884,AF394221,AY603654,AY839588,AY839589,AY839590,DQ176636, EU068667,EU074034,HM147822,HM147823,HM147824,KJ131500,KJ131501,KJ131502, KJ131503,KJ131504

Appendix figure 3. Results of West Nile virus Lineage 1A phylogenetic tree root-to-tip regression

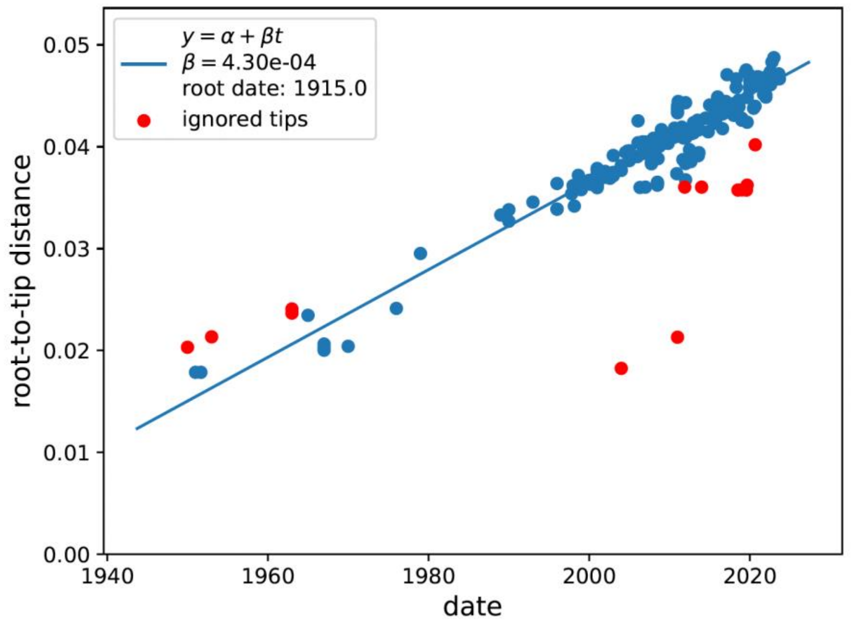

Appendix figure 4. Results of West Nile virus Lineage 2 phylogenetic tree root-to-tip regression

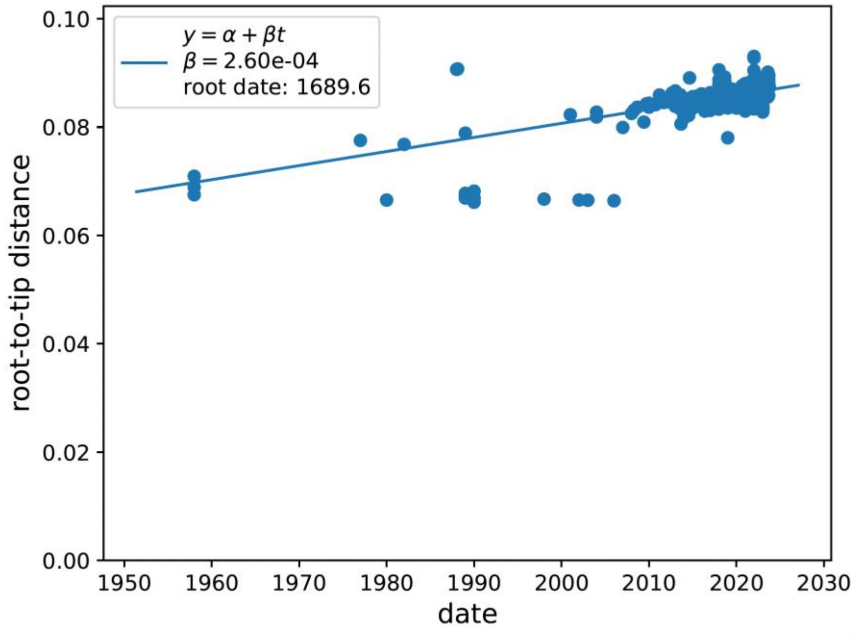

Detailed methodology for phylogenetic analyses

We retrieved whole genome sequences from NCBI Virus (https://www.ncbi.nlm.nih.gov/labs/virus/vssi/#/) on 13 August 2024 with a filter of 10 kilobase (kb) minimum sequence length. We retrieved sequences from all host and vector species. We subsampled sequences from the United States of America with the NCBI Virus function of randomly selecting three sequences per sample collection year (n = 66). We manually screened sequences for quality to confirm <10% base ambiguity and removed sequences lacking collection date and sampling locations. We analysed sequences through Genome Detective West Nile Virus Typing Tool for lineage assignment (33). We selected only Lineage 1A (L1A) and Lineage 2 (L2) sequences for further analysis as these are the most prevalent lineages in Africa (18). Lineage specific alignments were performed in MAFFTv7 (34). We utilised RDP5.59 to perform a full recombination screening with the two alignments to identify recombinant sequences (35). We identified six recombinants that were removed from alignments for further analyses (OP734266, OP846974, MF797870 for L1A and OK239667, EF429199, OQ214888 for L2). Additionally, we removed sequences of highly passaged isolates or clonal variants. The final alignments contained 258 and 541 sequences for Lineage 1A and Lineage 2, respectively.

We reconstructed phylogenetic trees and phylogeographic dispersal separately for L1A and L2 whole genome alignments. We constructed maximum likelihood phylogenies with IQTree v2.3.6 (36) including a search for best-fitting models with ModelFinder Plus (L1A was GTR+F+I+R3 according to Bayesian Information Criterion; and GTR+F+I+R4 for L2) and 1,000 bootstrap approximations. We inspected the tree topologies for temporal molecular clock signals using the clock functionality of TreeTime (37), removed outlier sequences (n=2 for L1A and n=8 for L2) that deviated from the strict molecular clock assumption, and created time-scaled phylogenies using adjusted mutation rates for each lineage (**β**=4.35 ╳ 10-4 for L1A and **β**=2.62 ╳ 10-4 for L2) as determined in a root-to-tip regression analysis (Supplementary information S4 and S5). We ran the mugration model with the time-scaled phylogenies in TreeTime to annotate discrete country locations to tips and infer country locations for internal nodes. Geographical resolution was at the country level for all sequences. Using a custom python script, we quantified the state changes over the phylogenies from the roots toward the tips. Visualisation and annotation of phylogenetic trees and viral exchange mapping were done in R. The maps link the origin to the destination country with curved lines going anti-clockwise in the direction of the curve. Each curved line is coloured by the mean date of state changes occurring along that specific link.

Appendix table 2. West Nile Virus isolations from mosquito and tick vector species per African country. Sub-national location of sample collection is provided or otherwise blank if the information could not be retrieved from the primary study. † denotes where taxonomic identification to species level was not provided. Minimum infection rate (MIR) and 95% confidence intervals (CI) are provided if available from the primary study. We calculated the MIR (74) if the primary study did not and the relevant information was provided. These values are denoted with an asterisk (*). Study type category is provided for each study.

**Country**	**Sub-national location**	**Vector species**	**Minimum infection rate (95% CI)**	**Year of sample collection**	**Study type**	**Reference**
Algeria	Aougrout	*Culex perexiguus*	0.56*	2015	Research	(1)
Algeria	-	*Culex species*†	-	1968	Research	(2)
Central African Republic	-	*Culex species*†	-	1967	Research	(2)
Central African Republic	-	*Culex tigripes*	-	1972	Research	(2)
Djibouti	Djibouti City	*Culex pipiens ssp. torridus*	1.49*	2010-2012	Indicator-based	(3)
Djibouti	Djibouti City	*Culex quinquefasciatus*	0.89*	2010-2012	Indicator-based	(3)
Egypt	-	*Culex antennatus*	5.00	1993	Event-based	(4)
Egypt	-	*Culex perexiguus*	23.00	1993	Event-based	(4)
Egypt	-	*Culex pipiens*	2.00	1993	Event-	(4)

					based	
Egypt	Kafr Elsheikh	*Culex species*[†]	2.00*	2019-2020	Research	(5)
Ivory Coast	-	*Culex guiarti*	-	1981	Research	(2)
Kenya	-	*Aedes albothorax*	-	-	Research	(2)
Kenya	Ijara district	*Amblyomma gemma*	1.91*	2010-2012	Research	(6)
Kenya	Northwest Kenya	*Culex univittatus*	1 individual (of 4) tested positive	1998	Event-based	(7)
Kenya	Ijara district, Gumarey	*All mosquito species pooled*	1.40 (0.39-3.30)	2006-2007	Research	(8)
Kenya	Ijara district, Sogan-Godud	*All mosquito species pooled*	7.50 (1.4-24)	2006-2007	Research	(8)
Kenya	Ijara district	*Rhipicephalus pulchellus*	0.25*	2010-2012	Research	(6)
Madagascar	Marofondroboka, Mitsinjo district	*Aedeomyia madagascarica*	-	2012-2013	Research	(9)
Madagascar	Marofondroboka, Mitsinjo district	*Anopheles coustani*	-	2012-2013	Research	(9)
Madagascar	Antsalova district	*Anopheles pauliani*	-	2012-2013	Research	(9)
Madagascar	-	*Culex quinquefasciatus*	-	1986	Research	(2)
Madagascar	-	*Culex univittatus*	-	1988	Research	(2)
Madagascar	Antsalova district	*Mansonia uniformis*	-	2012-2013	Research	(9)
Namibia	Mashi Conservancy, Zambezi region	*Culex univittatus*	0.98*	2018	Research	(10)
Niger	Tasnala	Sandflies[†]	0.01	2016	Event-based	(11)
Senegal	Barkedji	*Aedes dalzieli*	0.70	2012-2013	Indicator-based	(12)
Senegal	Barkedji	*Aedes dalzieli*	0.70	2012-2013	Indicator-based	(12)
Senegal	Barkedji	*Aedes vexans*	-	2006-2016	Research	(13)
Senegal	-	*Aedes vexans*	-	1990	Research	(2)
Senegal	Barkedji	*Anopheles rufipes*	10.00	2012-2013	Indicator-based	(12)
Senegal	Barkedji	*Anopheles rufipes*	10.00	2012-2013	Indicator-based	(12)
Senegal	Thille Boubacar	*Culex antennatus*	-	2006-2016	Research	(13)
Senegal	Barkedji	*Culex antennatus*	2.20	2012-2013	Indicator-based	(12)
Senegal	Barkedji	*Culex antennatus*	2.20	2012-2013	Indicator-based	(12)
Senegal	Barkedji	*Culex bitaeniorhynchus*	-	2006-2016	Research	(13)
Senegal	Barkedji	*Culex neavei*	-	2006-2016	Research	(13)
Senegal	-	*Culex neavei*	-	1993	Research	(2)
Senegal	Matam	*Culex neavei*	-	2017	Indicator-based	(14)
Senegal	Barkedji	*Culex neavei*	5.80	2012-2013	Indicator-based	(12)

Senegal	Barkedji	*Culex neavei, Cx. Poicilipes, Cx. ethiopicus*	-	2016	Indicator-based	(14)
Senegal	Barkedji	*Culex neavei*, *Cx. tritaeniorhynchus*	-	2013	Indicator-based	(14)
Senegal	Barkedji	*Culex perfuscus*	-	2006-2016	Research	(13)
Senegal	Barkedji	*Culex perfuscus*	1.80	2012-2013	Indicator-based	(12)
Senegal	Barkedji	*Culex perfuscus*	1.80	2012-2013	Indicator-based	(12)
Senegal	Barkedji	*Culex poicilipes*	-	2006-2016	Research	(13)
Senegal	-	*Culex poicilipes*	-	1990	Research	(2)
Senegal	Barkedji	*Culex poicilipes*	0.10	2012-2013	Indicator-based	(12)
Senegal	Barkedji	*Culex quinquefasciatus*	-	2006-2016	Research	(13)
Senegal	Barkedji	*Culex quinquefasciatus*	12.50	2012-2013	Indicator-based	(12)
Senegal	Barkedji	*Culex quinquefasciatus*	12.50	2012-2013	Indicator-based	(12)
Senegal	Barkedji	*Culex quinquefasciatus*, *Cx. perfuscus*, *Cx. neavei*, *Cx. poicilipes*, *Cx. tritaeniorhynchus*, *Cx. antennatus*, *Anopheles rufipes* and *Aedes dalzieli*	-	2012	Indicator-based	(14)
Senegal	Barkedji	*Culex tritaeniorhynchus*	0.60	2012-2013	Indicator-based	(12)
Senegal	Barkedji	*Culex tritaeniorhynchus*	0.60	2012-2013	Indicator-based	(12)
Senegal	-	*Mimomyia lacustris*	-	1990	Research	(2)
South Africa	Bethulie, Free State	*Aedes caballus*	-	1969	Research	(15)
South Africa	Lapalala	*Anopheles spp*†, *Aedes spp*†, *Culex univittatus*	1.01	2011-2018	Indicator-based	(16)
South Africa	Boschkop	*Culex pipiens, Cx. univittatus*	0.72	2011-2018	Indicator-based	(16)
South Africa	Kyalami	*Culex pipiens*, *Cx. univittatus, Cx. theileri*	2.53	2011-2018	Indicator-based	(16)
South Africa	Marakele	*Culex poicilipes, Anopheles gambiae s.l.*	0.33	2011-2018	Indicator-based	(16)
South Africa	Kruger National Park	*Culex simpsoni*, *Cx. perexiguus*, *Cx. bitaeniorhynchus*	1.23	2011-2018	Indicator-based	(16)
South Africa	Olifantsvlei, Gauteng	*Culex theileri*	-	1962	Research	(15)
South Africa	Welkom, Free State	*Culex univittatus*	-	1963	Research	(15)
South Africa	Bethulie, Free State	*Culex univittatus*	-	1969	Research	(15)
South Africa	Rondebult, Gauteng	*Culex univittatus*	-	1984	Research	(15)

South Africa	Olifantsvlei, Gauteng	*Culex univittatus*	-	1962, 1963, 1964, 1965, 1966	Research	(15)
Tunisia	Saddaguia and El Felta	*Culex pipiens*	6.80	2014	Event-based	(17)
Tunisia	Khniss	*Culex pipiens*	14.93	2011	Event-based	(17)
Tunisia	Agba	*Culex pipiens*	1 individual (of 19) tested positive	2012	Event-based	(17)
Tunisia	Moôtmar- Sahline	*Culex pipiens*	1 individual (of 9) tested positive	2012	Event-based	(17)
Tunisia	Oued khniss	*Culex pipiens*	8.00*	2012	Event-based	(17)
Zambia	Mongu district	*Culex quinquefasciatus*	3.62 (0.21-17.85)	2012-2016	Research	(18)

Appendix figure 5. A) Point locations (blue) of samples screened by studies with polymerase chain reaction (PCR) methods. Dark grey colouring portrays the countries from which samples were collected. Gabon is zoomed in for resolution of sample locations in the side panel; B) Point locations (gold) of samples collected for genomic sequencing. Tunisia and South Africa are zoomed in on the side panels

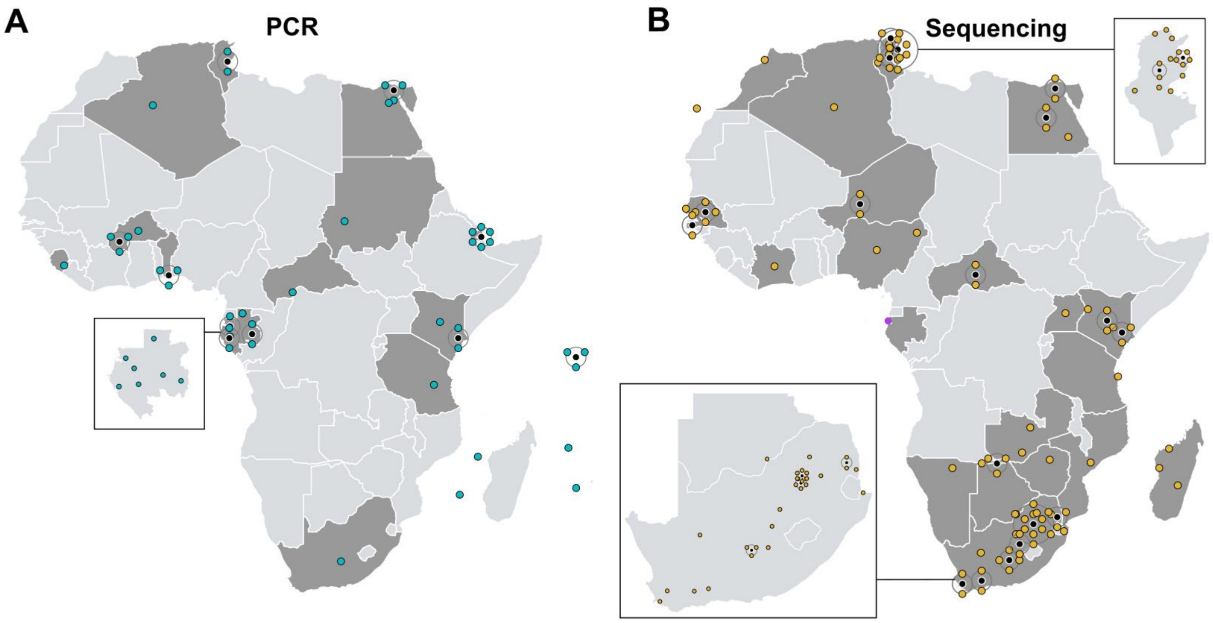

Appendix figure 6. Temporal distribution of genomic publications included in the review (grey squares) per country as well as the collection date and the number of publicly available sequences shown by orange circles.

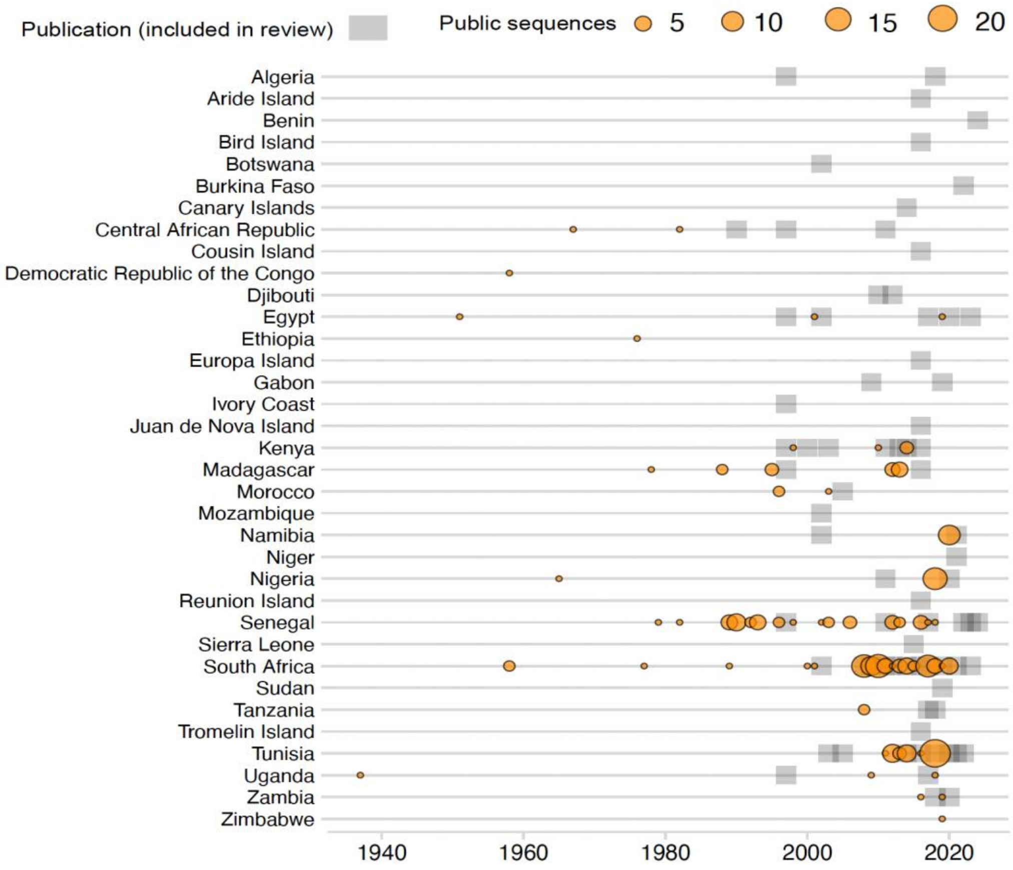

Appendix figure 7. Global and African genetic diversity of West Nile Virus. A) Log transformed number of whole genomes per geographic region (retrieved from NCBI on 27 August 2024); B) Proportion of complete genomes per lineage of West Nile virus distributed over time from a global genomic dataset; C) Temporal distribution of proportions of complete genomes per lineage collected from Africa; D) Spatial occurrences of West Nile virus lineages in Africa from complete and partial genomes.

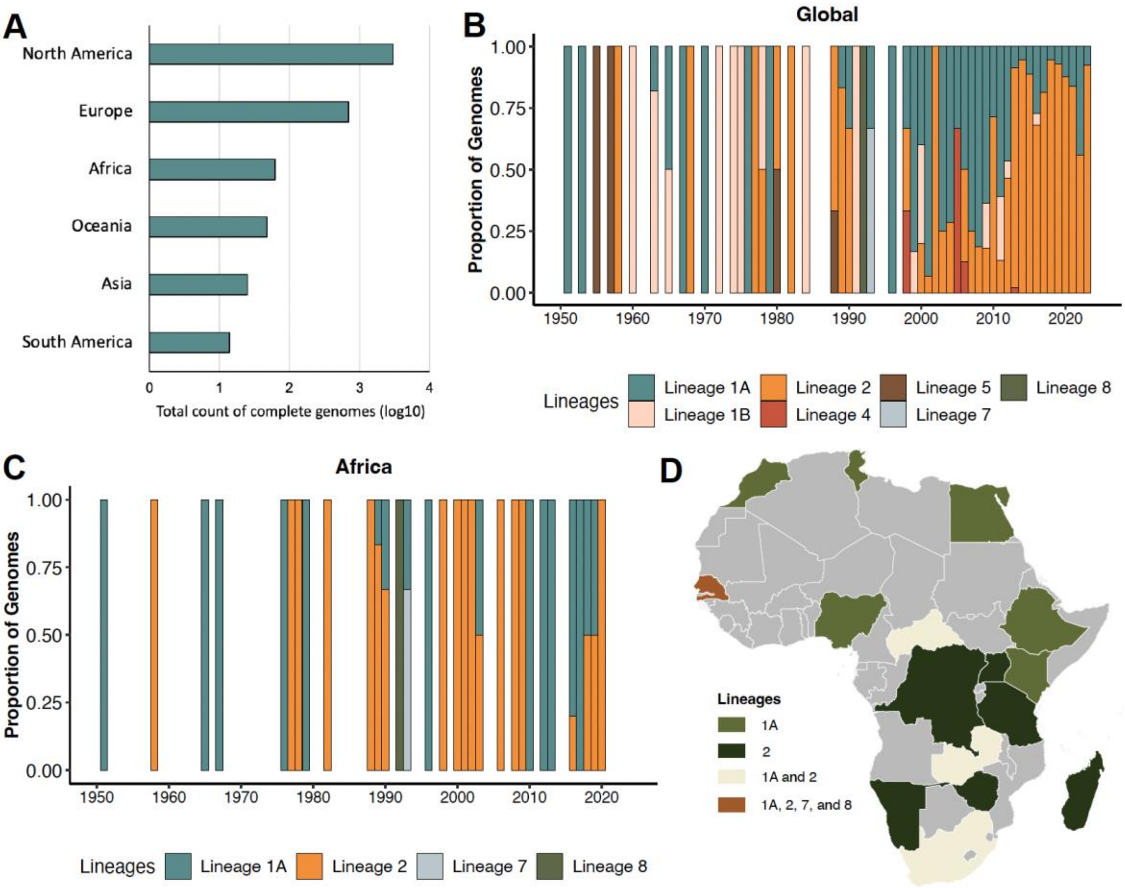

Appendix figure 8. For studies that produced African genomic data, the large pie chart depicts the proportion of publications that performed molecular laboratory protocols internally (i.e., within the African country where samples were collected) or externally (i.e., sent to another laboratory). The smaller pie chart displays the locations for studies that employed external laboratory services.

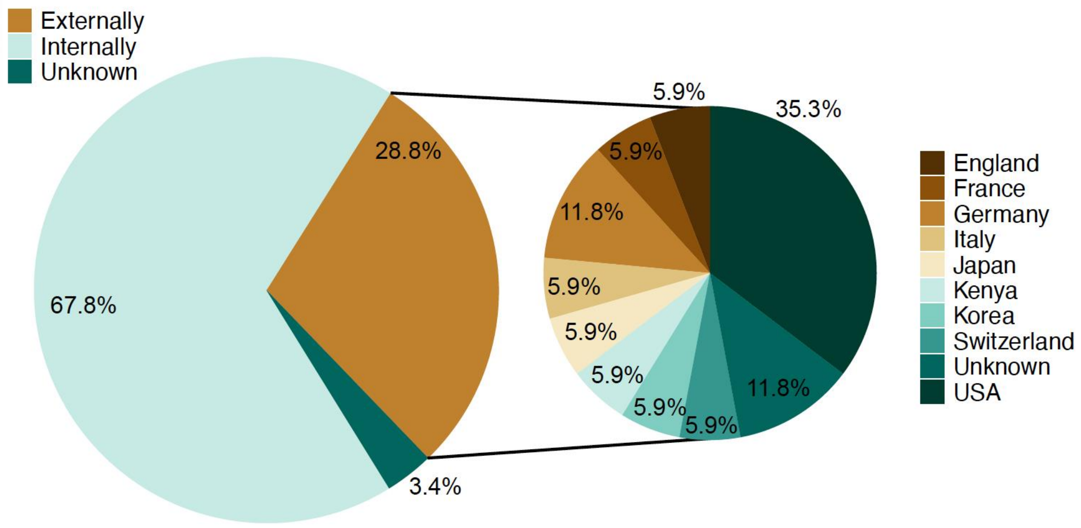

Appendix figure 9. Number of publications that employed molecular methods of polymerase chain reaction (PCR) (solely) or sequenced genomic regions per study type.

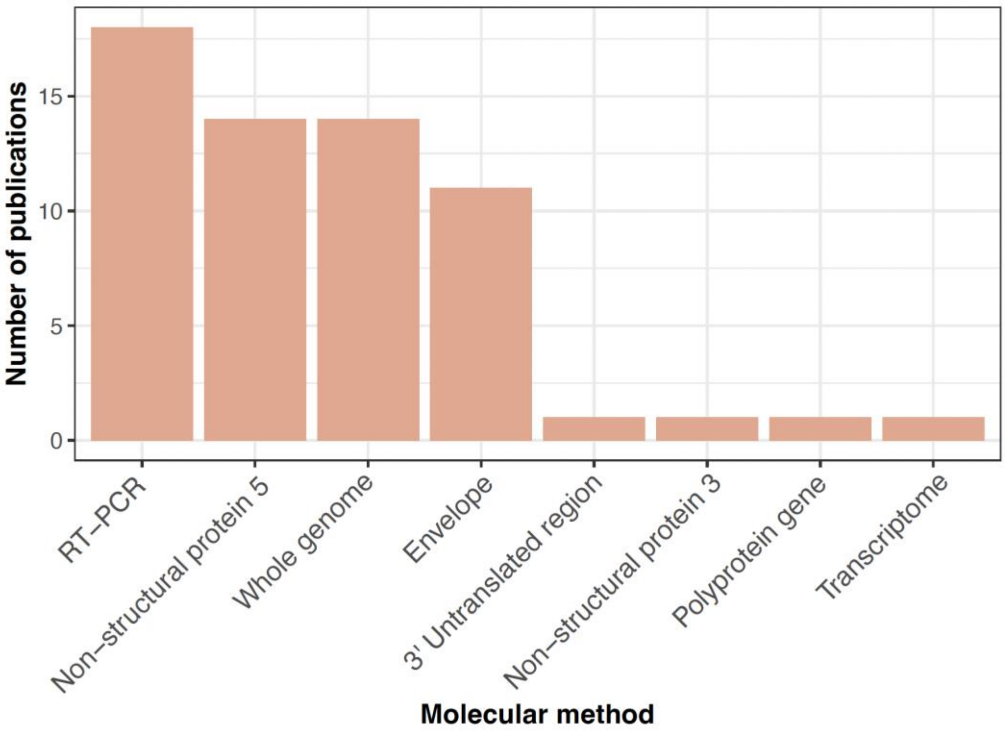

Appendix figure 10. Temporal progression of the use of different sequencing methods summed by publications per year.

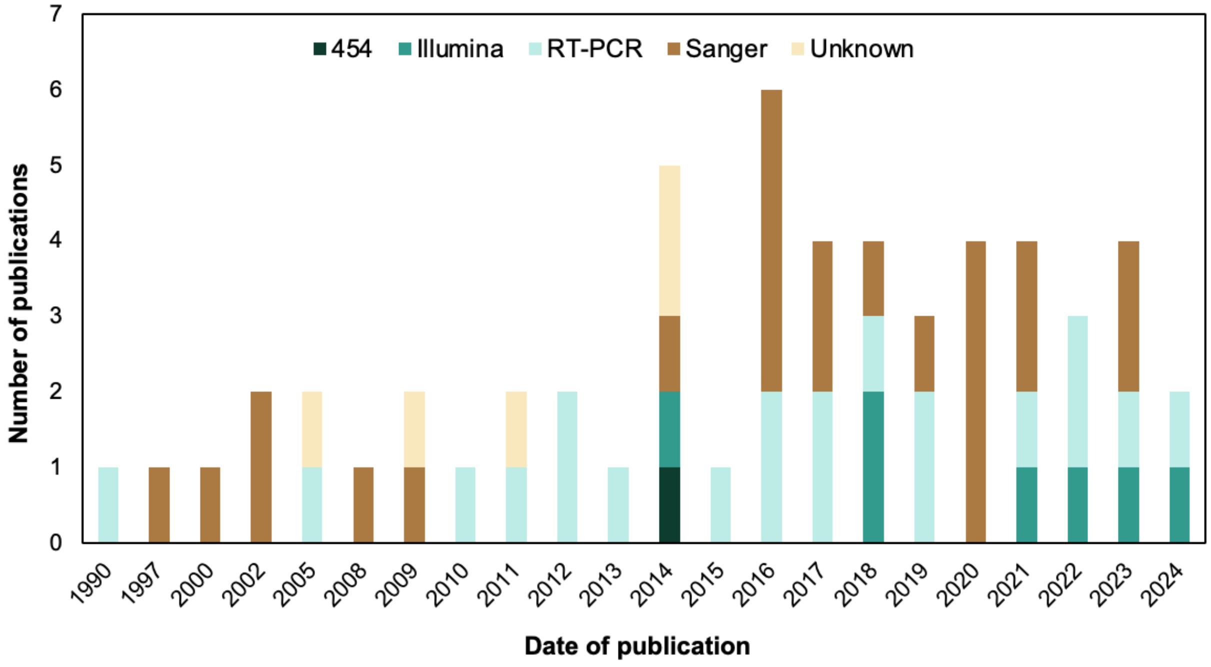

References

1. Benbetka S, Hachid A, Benallal KE, Benbetka C, Khaldi A, Bitam I, et al. First field evidence infection of Culex perexiguus by West Nile virus in Sahara Oasis of Algeria. J Vector Borne Dis. 2018;55(4):305–9.

2. Berthet FX, Zeller HG, Drouet MT, Rauzier J, Digoutte JP, Deubel V. Extensive nucleotide changes and deletions within the envelope glycoprotein gene of Euro-African West Nile viruses. J Gen Virol. 1997 Sep;78 (Pt 9):2293–7.

3. Faulde MK, Spiesberger M, Abbas B. Sentinel site-enhanced near-real time surveillance documenting West Nile virus circulation in two Culex mosquito species indicating different transmission characteristics, Djibouti City, Djibouti. J Egypt Soc Parasitol. 2012 Aug;42(2):461–74.

4. Turell MJ, Morrill JC, Rossi CA, Gad AM, Cope SE, Clements TL, et al. Isolation of west nile and sindbis viruses from mosquitoes collected in the Nile Valley of Egypt during an outbreak of Rift Valley fever. J Med Entomol. 2002 Jan;39(1):248–50.

5. Selim A, Radwan A, Arnaout F. Seroprevalence and molecular characterization of West Nile Virus in Egypt. Comp Immunol Microbiol Infect Dis. 2020 Mar 24;71:101473.

6. Lwande OW, Lutomiah J, Obanda V, Gakuya F, Mutisya J, Mulwa F, et al. Isolation of tick and mosquito-borne arboviruses from ticks sampled from livestock and wild animal hosts in Ijara District, Kenya. Vector Borne Zoonotic Dis. 2013 Sep;13(9):637–42.

7. Miller BR, Nasci RS, Godsey MS, Savage HM, Lutwama JJ, Lanciotti RS, et al. First field evidence for natural vertical transmission of West Nile virus in Culex univittatus complex mosquitoes from Rift Valley province, Kenya. Am J Trop Med Hyg. 2000 Feb;62(2):240–6.

8. LaBeaud AD, Sutherland LJ, Muiruri S, Muchiri EM, Gray LR, Zimmerman PA, et al. Arbovirus prevalence in mosquitoes, Kenya. Emerging Infect Dis. 2011 Feb;17(2):233–41.

9. Maquart M, Boyer S, Rakotoharinome VM, Ravaomanana J, Tantely ML, Heraud J-M, et al. High prevalence of west nile virus in domestic birds and detection in 2 new mosquito species in madagascar. PLoS ONE. 2016 Jan 25;11(1):e0147589.

10. Guggemos HD, Fendt M, Hieke C, Heyde V, Mfune JKE, Borgemeister C, et al. Simultaneous circulation of two West Nile virus lineage 2 clades and Bagaza virus in the Zambezi region, Namibia. PLoS Negl Trop Dis. 2021 Apr 2;15(4):e0009311.

11. Fall G, Diallo D, Soumaila H, Ndiaye EH, Lagare A, Sadio BD, et al. First detection of the west nile virus koutango lineage in sandflies in niger. Pathogens. 2021 Feb 24;10(3).

12. Ndiaye EH, Diallo D, Fall G, Ba Y, Faye O, Dia I, et al. Arboviruses isolated from the Barkedji mosquito-based surveillance system, 2012-2013. BMC Infect Dis. 2018 Dec 12;18(1):642.

13. Mencattelli G, Ndione MHD, Silverj A, Diagne MM, Curini V, Teodori L, et al. Spatial and temporal dynamics of West Nile virus between Africa and Europe. Nat Commun. 2023 Oct 13;14(1):6440.

14. Ndione MHD, Ndiaye EH, Faye M, Diagne MM, Diallo D, Diallo A, et al. Re-Introduction of West Nile Virus Lineage 1 in Senegal from Europe and Subsequent Circulation in Human and Mosquito Populations between 2012 and 2021. Viruses. 2022 Dec 6;14(12).

15. Burt FJ, Grobbelaar AA, Leman PA, Anthony FS, Gibson GVF, Swanepoel R. Phylogenetic relationships of southern African West Nile virus isolates. Emerging Infect Dis. 2002 Aug;8(8):820–6.

16. MacIntyre C, Guarido MM, Riddin MA, Johnson T, Braack L, Schrama M, et al. Survey of West Nile and Banzi Viruses in Mosquitoes, South Africa, 2011-2018. Emerging Infect Dis. 2023 Jan;29(1):164–9.

17. Monastiri A, Mechri B, Vázquez-González A, Ar Gouilh M, Chakroun M, Loussaief C, et al. A four-year survey (2011-2014) of West Nile virus infection in humans, mosquitoes and birds, including the 2012 meningoencephalitis outbreak in Tunisia. Emerg Microbes Infect. 2018 Mar 14;7(1):28.

18. Orba Y, Hang'ombe BM, Mweene AS, Wada Y, Anindita PD, Phongphaew W, et al. First isolation of West Nile virus in Zambia from mosquitoes. Transbound Emerg Dis. 2018 Aug;65(4):933–8.
